# ACE-Net: A-line coordinates encoding network for vascular structure segmentation in ultrasound images

**DOI:** 10.1007/s11517-025-03323-z

**Published:** 2025-04-02

**Authors:** Beatriz Farola Barata, Guiqiu Liao, Gianni Borghesan, Keir McCutcheon, Johan Bennett, Benoit Rosa, Michel de Mathelin, Florent Nageotte, Michalina J. Gora, Jos Vander Sloten, Emmanuel Vander Poorten, Diego Dall’Alba

**Affiliations:** 1 Robot-Assisted Surgery Group, Department of Mechanical Engineering, KU Leuven, Leuven, 3001 Belgium; 2 Altair Robotics Laboratory, Department of Computer Science, University of Verona, Verona, 37134 Italy; 3 ICube, University of Strasbourg-CNRS-Inserm, UMR 7357, Strasbourg, France; 4 Flanders Make@KU Leuven, Leuven, 3000 Belgium; 5 Department of Cardiovascular Diseases, UZ Leuven, Leuven, 3000 Belgium; 6 Department of Cardiovascular Sciences, KU Leuven, Leuven, 3000 Belgium; 7 Wyss Center for Bio and Neuroengineering, Campus Biotech, Chemin des Mines 9, Geneva, 1202 Switzerland

**Keywords:** Computer aided intervention, Convolutional neural network, Coordinates regression, Deep learning, Image contour segmentation, Ultrasound imaging

## Abstract

**Abstract:**

Ultrasound (US) imaging enables the evaluation of vascular structures in real time, and it can provide morphological and pathological information during US-guided procedures. Automatic prediction of vascular structure boundaries can help clinicians in locating and measuring target structures more accurately and efficiently. Most existing US segmentation methods use per-pixel classification or regression, which require post-processing to obtain contour coordinates. In this work, we present ACE-Net, a novel approach that directly predicts the contour coordinates for every scanning line (A-line) in US images. ACE-Net combines two main modules: a boundary regression module that predicts the upper and lower coordinates of the target area for each A-line, and an A-line classification module that determines whether an A-line belongs to the target area or not. We evaluated our method on three clinical US datasets using, among others, dice similarity coefficient (DSC) and inference time as performance metrics. Our method outperformed state-of-the-art segmentation methods in inference time while achieving superior or comparable performance in DSC. ACE-Net is publicly available at https://github.com/bfarolabarata/ace-net.

**Graphical abstract:**

ACE-Net is a deep CNN architecture designed for direct contour segmentation of vascular structures in ultrasound images. It formulates segmentation as a boundary regression process combined with the prediction of vascular structure presence. To achieve this, ACE-Net introduces an A-line-based encoding scheme, representing contour coordinates of each vascular structure as two boundary vectors (upper and lower). The network consists of a backbone module, an A-line feature extractor, a multi-scale fusion module, and two final head encoders, which output boundary coordinates and presence probabilities. By leveraging a presence vector, ACE-Net enhances segmentation accuracy and effectively handles common ultrasound artifacts without requiring pre-processing. Its architecture is specifically designed to address the imbalance between lateral and axial information in ultrasound images, and it enables real-time clinical metric computation.

**Figure:**
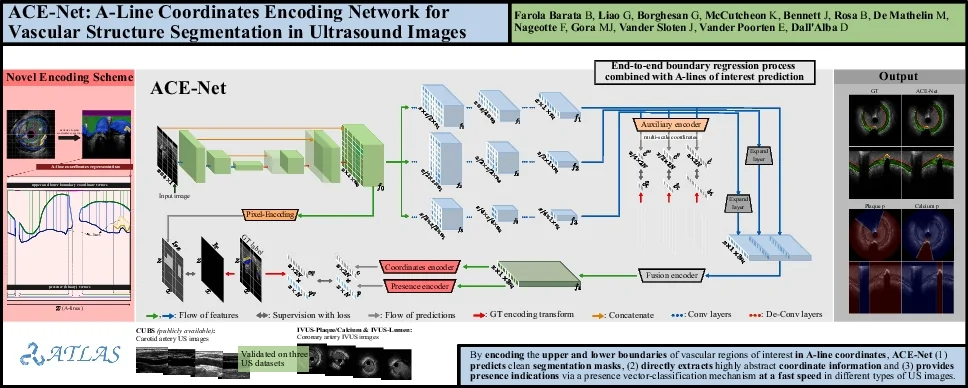

**Supplementary Information:**

The online version contains supplementary material available at https://doi.org/10.1007/s11517-025-03323-z.

## Introduction

Cardiovascular diseases are the primary cause of death worldwide, reported to be responsible for 32% of deaths globally in 2019 [1] and with coronary artery disease being responsible for about one third of these deaths [2]. Ultrasound (US) imaging plays an increasingly important role in both diagnosis and treatment of these conditions. For example, Lekakis et al. investigated the use of B-mode US to measure carotid intima-media thickness (IMT) as an indicator of the extent and severity of coronary atherosclerosis [3]. Similarly, intravascular ultrasound (IVUS) imaging is commonly used to obtain metrics related to the presence and extent of calcification (e.g., calcification angle), which can be used to assess both coronary atherosclerotic plaque progression and the risk of adverse cardiovascular events [4]. IVUS is also increasingly used during percutaneous coronary intervention (PCI), where it provides real-time pathological and morphological cross-sectional information of intracoronary structures such as atherosclerotic plaque. This information has the potential to improve PCI outcomes [5]. Reports further indicate that IVUS shows higher sensitivity and specificity detecting calcium deposits compared to other imaging modalities such as angiography and optical coherence tomography (OCT) [4].

Real-time automatic segmentation of vascular structures in US images has the potential to significantly improve the diagnosis and morphological characterization of coronary artery disease, especially during IVUS-guided PCI performed by operators with limited experience [4, 5]. Furthermore, a real-time segmentation technique that directly outputs contour position information could facilitate PCI navigation by supporting the prompt computation of precise quantitative pathological metrics and the intra-operative estimation of tool-tissue interaction and relative distances.

Nonetheless, existing segmentation methods for US applications are primarily designed to perform per-pixel classification or regression of grayscale B-mode images, which predicts regions of interest (ROIs) masks [6–10]. If one needs a vector that contains explicit coordinates of a contour, a post-processing step is necessary to extract them from the ROIs masks. However, this step can be challenging in certain areas, such as those affected by image artifacts or where vascular structures overlap. This is because even a few incorrectly classified pixels can introduce errors during the post-extraction of boundary coordinate vectors [11].

In this work, we propose a novel deep convolutional neural network (CNN) architecture, termed *A-line coordinates encoding network (ACE-Net)*, that targets direct contour segmentation of vascular structures. Motivated by approaches that segment ROIs by predicting polygons or contours together with bounding boxes [12, 13], this work formulates the segmentation task as a boundary regression process coupled with ROI presence prediction. We exploit the axial information of B-mode US images, whereby a one-dimensional vector in an image axial direction is defined as an A-line. Following this approach, a novel A-line-based encoding scheme that directly encodes contour coordinates of any target ROI as a set of two boundary vectors (upper and lower coordinates) defined per A-line is introduced. Moreover, ACE-Net predicts in real time a presence vector containing the probability of relevant vascular structures being present for each A-line. This allows ACE-Net to better handle common US image artifacts (e.g., shadows, guidewire effects) that might cause discontinuities in contours, without the need of a pre-processing step. Conversely to conventional CNN-based approaches [9, 14, 15] that equally down-sample and up-sample images and feature maps in two dimensions, ACE-Net brings up a novel network architecture tailored to the proposed encoding scheme and designed to deal with significantly imbalanced lateral and axial information in US images. ACE-Net is evaluated on three US datasets. Results of this evaluation show that ACE-Net focuses on the contour location of target structures due to the proposed encoding scheme, and it outperforms both CNN and transformers-based state-of-the-art segmentation approaches.

In summary, the main *contributions* of this work are three-fold: (1) a fast, accurate, and easy to train network architecture, ACE-Net, which exploits the proposed A-line-based encoding scheme and can handle imbalanced lateral and axial image resolutions, while further directly outputting explicit coordinate information; (2) a novel A-line-based coordinates encoding scheme that represents the segmentation boundaries of ROIs as pairs of upper and lower contour coordinate vectors; (3) experimental evaluation on three segmentation tasks of carotid US and IVUS images. ACE-Net achieves state-of-the-art segmentation results when compared to CNN and transformers-based alternatives and outperforms them in terms of computational efficiency.

## Related work

In this section, an overview of relevant state-of-the-art approaches is provided. The section discusses localization of ROIs in generic and side-viewing images (Sect. 2.1), semantic segmentation of both non-medical and US images (Sect. 2.2), and lastly, existing shape encoding schemes designed for deep learning methods (Sect. 2.3).

### Target localization

One of the most widely used approaches for object localization in generic images is based on bounding box detection networks [16, 17]. Typically, bounding box models are designed to predict either an object’s height, width and center point, or the location of at least four outermost points. A bounding box can be considered as a high-level approximation of the contour of an object. Yet, it captures little information about an object’s shape beyond its location, scale, and aspect ratio [18]. Several other methods propose to predict key-points in order to detect objects [19–21]. For example, CornerNet [20] detects two bounding box corners as key-points, while ExtremeNet [19] detects the top-, left-, bottom-, right-, and center points of targets of interest. In order to increase the efficiency of object location representation, CenterTrack [21] has recently introduced an object center point probability regression model at every pixel of an image.

For side-viewing imaging modalities such as IVUS or OCT, one dimension (i.e., lateral scanning direction) localization approaches have been proposed to increase localization efficiency. Ughi et al. propose a non-learning-based A-line classification approach, which selects and locates ROIs in side-viewing OCT images, instead of classifying pixels to estimate the coverage of pathological areas [22]. This A-line localization approach was further explored by Kolluru et al., who develop a CNN-based approach for real-time plaque classification in coronary intravascular OCT images [23]. Also applied to intravascular OCT, a hybrid learning approach is explored in [24], which combined deep learning convolutional and hand-crafted, lumen morphological features for A-line classification. Nonetheless, even though A-line classification is a fast approach to detect the presence of a target in the lateral direction of side-viewing images, it does not estimate a target’s exact location in the axial direction. In contrast, ACE-Net is able to further improve localization accuracy by coupling coordinate regression with A-line classification.

### Semantic segmentation

The majority of image segmentation tasks can be identified as semantic segmentation, where per-pixel classification or regression is employed. Many approaches are based on CNNs [6]. For instance, FCN [14], Unet [15], and DeepLabV3 [9] realize pixel-to-pixel mapping using CNNs. More recent segmentation methods have adapted self-attention mechanisms from transformers [25, 26] to achieve pixel-wise classification [10, 27], which enhances the context role in comparison with conventional CNNs. In the field of vascular US, which includes the use of IVUS and external US, pixel-wise semantic approaches based on CNNs for the segmentation of intraluminal structures (e.g., lumen-intima layer, atherosclerotic plaque) have been investigated. Zhou et al. use a U-Net architecture to automatically segment carotid plaque in longitudinal carotid US images [7]. Mi et al. propose a multi-branch feature fusion network (MBFF-Net) that fuses multi-scale features to produce semantic segmentation of carotid plaque in US images [28]. For IVUS, Li et al. report an end-to-end architecture based on three modified U-Nets to simultaneously segment media-adventitia layers and luminal regions and locate calcified plaques [8]. Yang et al. propose IVUS-Net, a fully convolutional network (FCN)-based pipeline that predicts a pixel-wise mask followed by a contour extraction post-processing step to obtain luminal and vessel walls boundaries [29]. Bargsten et al. directly compare two CNN architectures: a U-Net with residual blocks and DeepLabV3 with a ResNet50 backbone to segment calcifications [9]. A similar approach is proposed by Blanco et al., in which a multi-frame CNN is followed by a Gaussian process regression-based contour extractor to segment only the lumen and vessel areas in IVUS sequences [30]. Despite their promising accuracy in region overlapping, all the aforementioned methods are sub-optimal in representing object boundaries and/or do not output boundary contours in an end-to-end approach, while ACE-Net provides an encoding scheme that allows for the direct regression of contour coordinates, in addition to achieving high segmentation accuracy.

### Shape encoding and prediction

Target contours are generally represented by polygon or boundary encoding approaches [12, 18]. Recently, several contour extraction techniques have been investigated to simultaneously provide direct target localization coordinates and semantic segmentation regions/masks. Castrejon et al. [12] deploy a recurrent neural network to predict the vertices of a polygon representing a target shape. In [18], the shape of objects is encoded with different representations coupled with a bounding box detection network that crops the target of interest. Moreover, Jetley et al. compare three encoding schemes: fixed-sized binary shape masks, a radial representation, and a learned shape encoding [18]. Following the radial representation encoding approach, where a series of offsets between an object’s anchor pixel and points on its contour are defined, Xu et al. predict Chebyshev approximation terms to regress the shape coordinates in the polar domain [31]. More recently, PolarMask [13] has been proposed, whereby two paralleled branches are used to first find the center-of-mass of a region and then regress a dense distance between the region’s center and its contour in polar coordinates. Unlike our proposed method, the aforementioned techniques are not pixel-accurate and assume that every object has a continuous contour, which is not always the case in US imaging, as image artifacts might cause contour discontinuities (e.g., during PCI, a guidewire could cast a shadow). Moreover, where PolarMask also couples detection and contour regression, ACE-Net achieves target detection by means of A-lines instead of bounding boxes and further integrates an efficient approach of encoding contour coordinates.

**Fig. 1:**
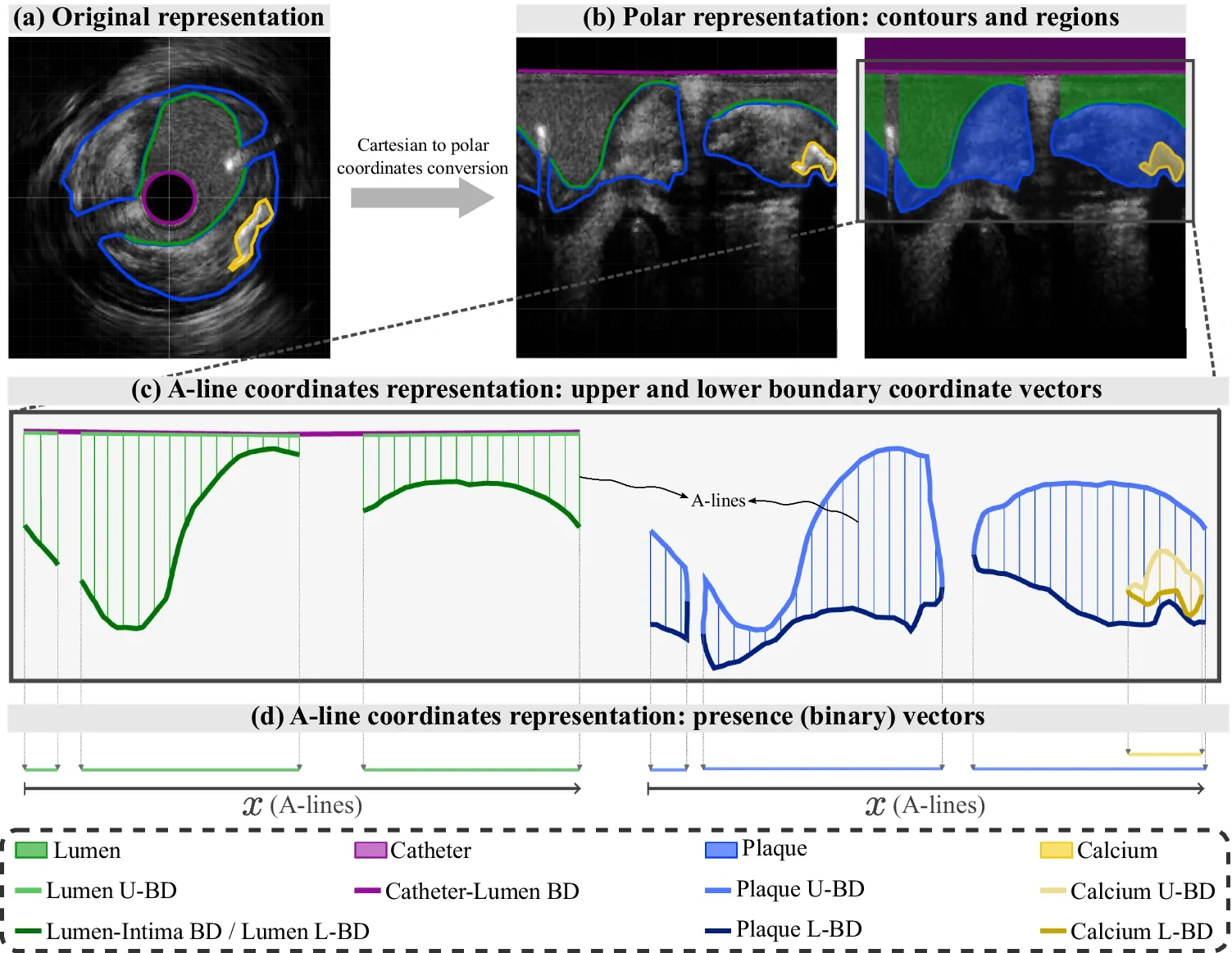
Illustration of the proposed encoding scheme. **a** and **b** are, respectively, the same IVUS image in its original (Cartesian domain) and polar representations. The polar representation shows the single boundary contour vectors (**b**—left) and the corresponding regions (**b**—right) for each vascular structure example. **c** describes the proposed A-line coordinates representation of example vascular target regions (lumen, plaque, and calcium) by a set of upper and lower boundaries defined per A-line. **d** depicts how the binary presence vectors (defined per A-line) for plaque, calcium, and lumen are determined based on the corresponding upper and lower boundary coordinate vectors. (BD: boundary; U-BD: upper boundary; L-BD: lower boundary)

## Methods

In this work, the segmentation task of vascular structures and/or layers is formulated as a boundary regression process in combination with A-lines of interest (AOIs) prediction. The proposed A-line coordinates-based encoding scheme for US images is first introduced in Sect. 3.1. Next, an overview of the ACE-Net architecture is detailed in Sect. 3.2, followed by comprehensive descriptions of its sub-modules. Lastly, the loss functions and training strategies are detailed in Sect. 3.3.

### A-line coordinates-based encoding scheme

The proposed A-line-based encoding scheme is devised for US images where A-lines are stacked in parallel. In this work, A-lines are defined as one-dimensional vectors in the axial direction of B-mode US images. Thus, for B-mode US imaging where linear external probes are used, images are directly handled by the proposed encoding scheme, as these are natively composed by A-lines stacked in parallel. Yet, in the case of IVUS, images obtained in a clinical setting typically show A-lines in a radial representation (Fig. 1a). These images are referred to as Cartesian domain IVUS images. Consequently, every pixel of the original IVUS frames and annotated contours (i.e., in the Cartesian domain) must first be converted to polar coordinates, as illustrated in Fig. 1b. To maintain a consistent notation throughout the rest of this paper, polar domain is used to refer to US images where A-lines are stacked in parallel, while Cartesian domain is used to refer to US images where A-lines are radially represented.

One polar domain image $I \in {{\mathbb {R}}^{{\displaystyle x}{\scriptstyle \times } {\displaystyle z}}}$ is composed by *x* A-lines, with *z* being the length of each A-line. For the polar domain images and contours, A-line coordinates are used to achieve a dense representation of the boundaries of structures of interest. That is, the shape of each target region type $n \in \{1,2,\dotsc , N\}$ is defined by upper and lower position boundaries, represented by a matrix with two rows $c_n \in {{\mathbb {R}}^{{\displaystyle x}{\scriptstyle \times } 2}}$, where one pair of elements $c_n^i \in {{\mathbb {R}}^2}$ denotes the upper and the lower position coordinates of target class *n* for A-line *i* (see Fig. 1c). *N* is the total number of ROI types with upper/lower boundaries. In addition, since target structures might not cover the entire polar image width (i.e., all A-lines), AOIs are discriminated from A-lines with no relevant information: $c_n^i$ is empty for each A-line *i* with no contour information of *n*. For every target ROI, $c_n$ is thus coupled with a presence probability vector, defined for all A-lines, $p_n \in {{\mathbb {R}}^{\displaystyle x}}$ (see Fig. 1d).

ACE-Net predicts all contours by extending the coordinates and AOI probability matrices as $c \in {{\mathbb {R}}^{{\displaystyle x}{\scriptstyle \times } 2N}}$ and $p \in {{\mathbb {R}}^{{\displaystyle x}{\scriptstyle \times } N}}$, respectively. Only coordinates with high presence probability values are considered as valid predictions.

**Fig. 2:**
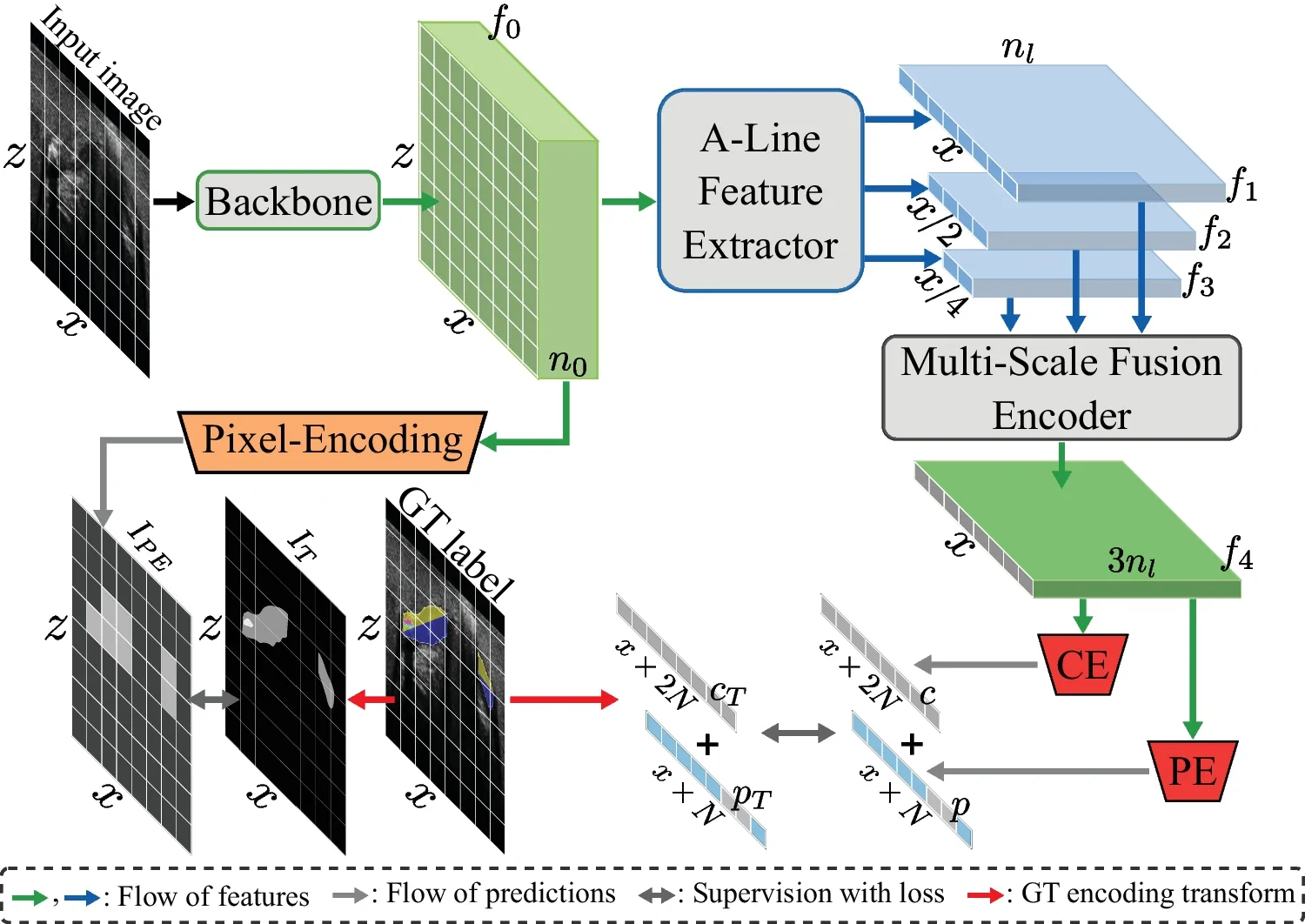
Diagram of the ACE-Net architecture designed to achieve A-line coordinates encoding. First, an A-line feature extractor follows a backbone to extract multi-scale features. Then, two encoders follow a fusion module to predict both the A-line coordinates and presence probability vectors. A multi-task learning strategy is deployed to train the backbone by using a pixel-encoder to predict pixel-wise segmentation. (CE, coordinates encoder; PE, presence probability encoder)

### ACE-Net architecture

#### Overview

The proposed ACE-Net is composed of a backbone module, an A-line feature extractor, a multi-scale feature fusion module, and two final head encoders that directly output boundary coordinates *c* and presence probabilities *p* (Fig. 2). All feature extractors and encoders are designed with CNNs, and no fully connected layers are used.

A polar domain input image is first fed into the backbone feature extractor which produces a high-resolution and semantically weak (i.e., low-level) feature map $f_0 \in {{\mathbb {R}}^{{\displaystyle x}{\scriptstyle \times } {\displaystyle z}{\scriptstyle \times } n_0}}$, with pixel-wise spatial correspondence. $n_0$ corresponds to the channel depth of $f_0$. $f_0$ is then processed by the A-line feature extractor that follows a parallel multi-scale encoding scheme. As a result, 3 levels of positioning features ${f}_{i},i=1,2,3$ are produced. A multi-scale fusion module is applied to re-organize the different levels of features, and the output ${f}_{4}$ is encoded with a coordinates encoder (CE) and a presence encoder (PE) to obtain the final outputs. While the two final encoders share the same concatenated input features ${f}_{4}$, they have different activation and training objective functions.

**Fig. 3:**
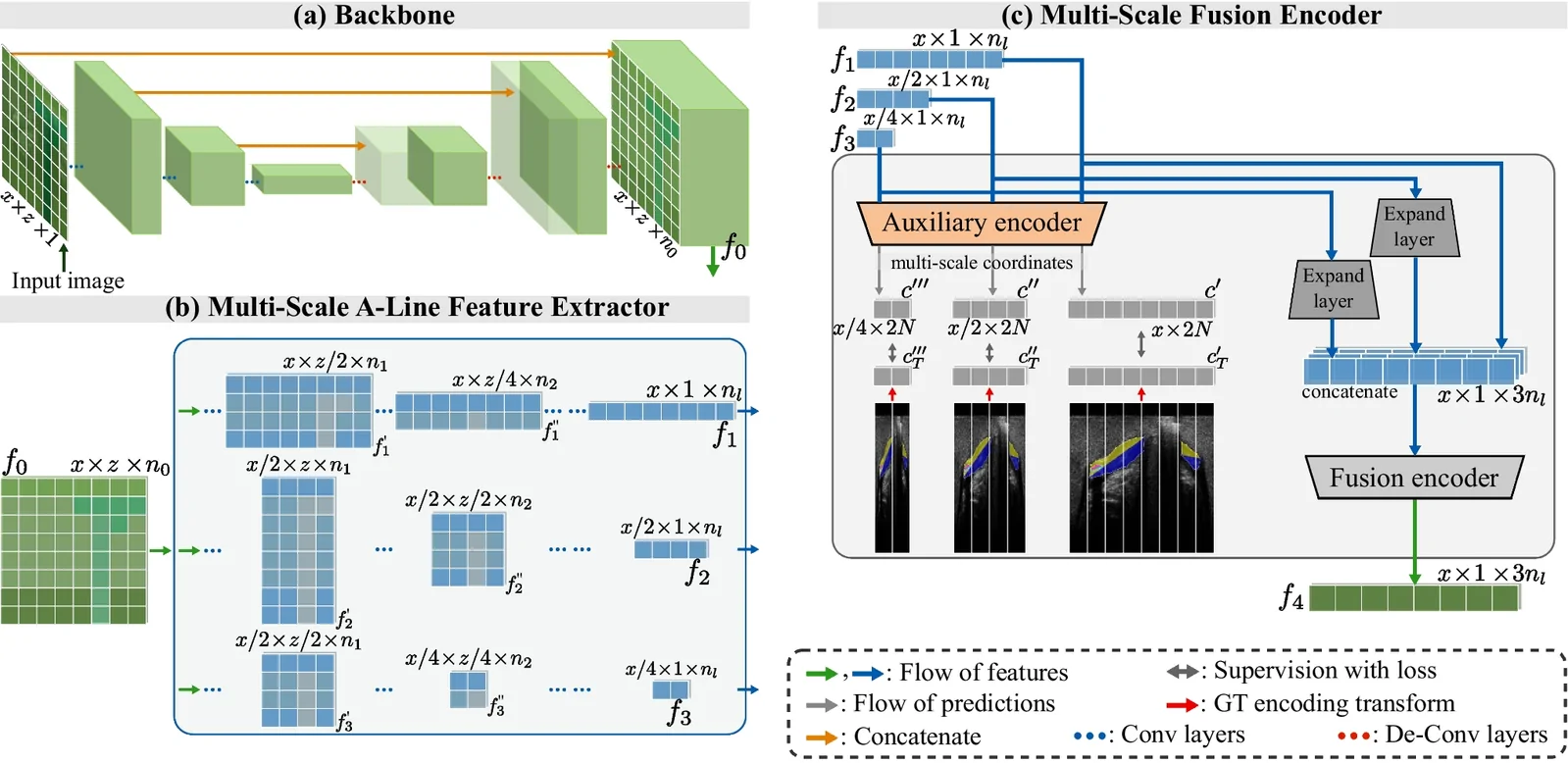
ACE-Net sub-modules architecture: **a** backbone feature extractor, which follows a down-sampling and up-sampling scheme to produce dense feature maps; **b** A-line feature extractor, which encodes A-line features as different horizontal scales to produce different spacial correspondences; and **c** multi-scale fusion encoder, where higher abstract features extracted by lower-scale branches are expanded to match image resolution and then fused with one final encoding layer. Features at different scales share the same auxiliary encoder to predict lower scale coordinates, and loss is computed with down-sampled ground truth. (GT, ground truth)

#### Backbone feature extractor and auxiliary pixel encoding

The backbone feature extractor is composed of a pyramid of down-sampling and up-sampling CNNs with shortcuts [32] (see Fig. 3a). Using the multi-task learning (MTL) technique, the backbone is trained with a pixel-wise loss. From the backbone, the feature map $f_0$ is processed by a pixel-encoder producing a pixel-wise map $I_{PE}$. This pixel-encoder is a $1\!\times \!1$ convolutional layer that reduces the channel depth of $f_0$ from $n_0$ to 1, thus enabling the backbone to learn spatial correspondences. Note that the backbone can be replaced with a simple ResNet block or even removed. The removal of the backbone is further discussed in the ablation study described in Sect. 5.5.

#### Multi-scale A-line feature extractor

Conversely to traditional segmentation methods that extract hierarchical information in a cascade of down-sampling and up-sampling [8, 29], the A-line feature extractor integrating ACE-Net follows a parallel multi-scale encoding scheme (Fig. 3b). This choice is done for two reasons: (1) to take advantage of the inherent difference in lateral and axial resolutions in US images and (2) to improve network inference efficiency [33]. All the convolutional layers in each branch have the same kernel size but different strides to control dimension reduction. In some branches, the strides are imbalanced in lateral/axial directions (e.g., the first branch never reduces the lateral dimension, but it reduces the axial dimension after each convolutional layer). As a result, the first order of abstract positioning features ${f}_{1}\in {{\mathbb {R}}^{{\displaystyle x}\times 1\times n_l}}$ matches the input image width x, where $n_l$ is the depth of *l*-th layer and proportional to the convolutional layer index *l* of the A-line feature extractor. In contrast, features ${f}_{2}$ and ${f}_{3}$ have lower spatial correspondence and a horizontal dimension size of ${\displaystyle x}/2$ and ${\displaystyle x}/4$, respectively.

#### Fusion encoder

A fusion encoder with auxiliary multi-scale predictions is introduced to reorganize the A-line features at different scales and address their axial/lateral imbalance. As shown in Fig. 3c, the multi-scale fusion module reorganizes the hierarchical coordinate position features. The fusion encoder follows two steps to combine information. First, expanding layers based on transposed convolution are used to up-scale features with lower horizontal scales (i.e., $f_2$ and $f_3$). Second, aligned features from different levels are concatenated and then fused with a convolution layer, providing the final feature map $f_4$. In addition, an auxiliary encoder (Fig. 3c), shared by all coordinate features, is added directly after each multi-scale feature $f_i$. Different scales of coordinate features are forced to represent features in a similar way (supervised by re-scaled ground truth (GT) $c_{T}'$, $c_{T}''$, and $c_{T}'''$) and maintain the hierarchy. All coordinate features ${f}_{i}$ are thus encoded to coordinate vectors corresponding to their lateral width, while avoiding assigning different individual auxiliary encoders. The shared auxiliary encoder is composed of $1\!\times \!3$ convolution layers that reduce the channel dimension to 2N, and it is only employed for training, while it is disabled during inference.

#### Coordinates and presence encoding modules

Similar to the auxiliary encoder, CE is composed of $1\!\times \!3$ convolution layers with linear activation, which reduce the channel dimension to 2N. In order to increase the segmentation accuracy, PE is applied to encode the final feature map $f_4$ to vectors p∈(0,1) with dimension ${\displaystyle x}\times N$. PE is a $1\!\times \!3$ convolution layer with sigmoid activation. By doing this, the presence probability *p* and the A-line coordinate vectors *c* are aligned for *N* types of objects at all A-line locations (see Fig. 2).

### Loss functions and training strategies

The ACE-Net training is divided into two stages: early training and fine-tuning, where different loss computation and network update are used. In the early training stage, supervised with a GT map $I_{T}$ obtained from the GT annotation, a pixel-wise semantic side-output image $I_{PE}$ (see Fig. 2) with a pixel encoding is enabled. Note that the subscript *T* is used to refer to GT information. Given that, in most cases, the coverage of one region class is smaller than the coverage of other regions to classify, a balanced distance loss is defined to optimize the backbone feature extractor as follows:

1 $$\begin{aligned} \mathcal {L}_I = \beta \sum _{j\in I^-_{T}} \parallel {I^j_{T}-I^j_{PE}}\parallel + (1-\beta ) \sum _{j\in I^+_{T}} \parallel {I^j_{T}-I^j_{PE}}\parallel \end{aligned}$$

where $\beta =|I^+_{T}|/|I_{T}|$, and $|I^+_{T}|$ and $|I^-_{T}|$ denote the sums of pixels within the ROI or not, respectively. For optimization of the top model of ACE-Net, which includes the A-line feature extractor and multi-scale fusion module, coordinate loss $\mathcal {L}_c$ and presence loss $\mathcal {L}_p$ are used. Both coordinates *c* and presence probability *p* are used to calculate the coordinate loss $\mathcal {L}_c$:

2 $$\begin{aligned} \mathcal {L}_c = \sum _{j=0}^{{\displaystyle x}N} (\parallel {c^{2j}_{T}-c^{2j}}\parallel + \parallel {c^{2j+1}_{T}-c^{2j+1}}\parallel ) p^j \end{aligned}$$

where $$c^{2j}$$ and $$c^{2j+1}$$ denote the upper and lower boundaries of a target region, respectively. It is worth mentioning that Eq. 2 is also used to calculate the auxiliary losses $$[\mathcal {L'}_c, \mathcal {L''}_c, \mathcal {L'''}_c]$$ for lower scale coordinates $\left[c^{\prime },c^{\prime \prime },c^{\prime \prime \prime }\right]$ from sub-feature maps.

The presence loss $\mathcal {L}_p$ is the cross entropy between binary GT and predicted presence probability vectors:

3 $$\begin{aligned} \mathcal {L}_p = -\sum _{j=0}^{{\displaystyle x}N}(p^j_{T}log(p^j)) + (1-p^j_{T})log(1-p^j) \end{aligned}$$

Note that in this work, during the early training stage, the backbone feature extractor is only optimized with $\mathcal {L}_I$, and the backbone layers are frozen for the backward propagation step with $\mathcal {L}_c$ and $\mathcal {L}_p$. Only in the subsequent fine-tuning stage, $\mathcal {L}_c$ and $\mathcal {L}_p$ are allowed to optimize the whole network. Effects of ablating parts of the losses and training strategies are discussed in Sect. 5.6.

The loss for the fine-tuning stage is a weighted loss:

4 $$\begin{aligned} \mathcal {L} = [\mathcal {L}_c, \mathcal {L}_{ca}, \mathcal {L}_p, \mathcal {L}_I]{\lambda }^T \end{aligned}$$

where $\mathcal {L}_{ca}$ is the average of multi-scale auxiliary loss $[\mathcal {L'}_c, \mathcal {L''}_c, \mathcal {L'''}_c]$. The weight setting ${\lambda } = [1, \ 1\!\times \!10^{-3}, \ 1\!\times \!10^{-3}, \ 1\!\times \!10^{-3}]$ is fixed. The specific number of loss weights and other training parameters can be found in the supplementary materials.

## Datasets

ACE-Net has been trained and evaluated on three datasets: CUBS, IVUS-plaque/calcium and IVUS-lumen. CUBS is a publicly available clinical dataset, whereas IVUS-plaque/calcium and IVUS-lumen were collected in-house, considering the unavailability of public datasets of IVUS-guided PCI.

### CUBS

CUBS [34] is a publicly available clinical dataset of common carotid artery US images acquired from both sides of the neck using an external US probe. It contains 2176 images, paired as left and right, from 1088 patients in 2 different centers. CUBS was collected to compare different measurement methods of common carotid IMT on the same database. The target of interest in CUBS is thus the intima-media region. In our work, this region is defined by upper and lower boundary coordinate vectors (of equal length) corresponding to the manually annotated lumen-intima and media-adventitia boundaries, respectively. The gold standard annotation, as mentioned in [34], was used as GT.

### IVUS-plaque/calcium

This dataset of IVUS images was collected between January and November 2021 at the University Hospitals (UZ) Leuven. IVUS images were acquired by two cardiologists from 10 chronic total occlusion (CTO) patients who underwent PCI (ages: 43–79; 8 males, 2 females; 5 patients showed severe calcifications: ≥ 50% reference lesion diameter). Note that CTOs are complete vessel occlusions present for at least 3 months due to obstructive atherosclerotic plaque [35]. All patients provided written informed consent to a protocol approved by the Ethics Committee Research UZ/KU Leuven (Study number S63611). The images were collected at a pullback speed of 1.0 *mm*/*s* using Opticross$^{\text {TM}}$ HD, 60MHz Coronary Imaging Catheters (Boston Scientific, MA, USA) with the POLARIS Multi-Modality Guidance System. From the 10 patients, a total of 1000 images (100 per patient) were included by manually selecting representative images containing atherosclerotic plaque and/or calcification areas. The annotation of plaque and calcium boundaries was performed using LabelMe [36] by one well-trained observer supervised by two expert cardiologists. No image artifacts were removed before manual annotation. All images were annotated in their original form (i.e., Cartesian domain), and each target region was segmented as a single boundary. All manual annotations were used as GT. Pre-processing was carried out to align the GT data with the A-line encoding scheme described in Sect. 3.1: images and annotated boundaries were first converted to their polar domain representations, and then each single contour was split into upper and lower vectors of coordinates by considering their local minimum and maximum A-line coordinates (see Fig. 1). The presence binary vectors were set to 1 for the A-lines where both the upper and lower coordinates vectors were defined.

### IVUS-lumen

The IVUS-lumen dataset contains 1000 images of vessels without severe calcifications and was extracted from the same study as the IVUS-plaque/calcium dataset (see Sect. 4.2). Similarly to the IVUS-plaque/calcium dataset, one well-trained observer, supervised by two experienced cardiologists, participated in annotating the data. For all images, lumen-intima and IVUS catheter-lumen boundaries were independently manually annotated in their Cartesian domain. In the pre-processing stage, after converting the manual annotations to their polar domain, the lumen-intima and catheter-lumen boundaries were, respectively, set as the lower and upper contours of the vessel lumen region. Note that since the IVUS catheter-lumen boundary is always defined for all A-lines (see Fig. 1), the presence probability (binary) vector of the vessel lumen region was set to 1 only for the A-lines where the lumen-intima contour is represented.

## Experiments and results

This section describes the extensive validation of ACE-Net. The evaluation metrics are first defined (Sect. 5.1), followed by the implementation details (Sect. 5.2). Next, the manual segmentation variability assessment carried out on the IVUS-plaque/calcium dataset is presented (Sect. 5.3). Following, a state-of-the-art comparison study (Sect. 5.4) and an ablation study (Sect. 5.5) are detailed. Evaluation on the effect of multi-task learning and freezing strategy (Sect. 5.6), unseen patients (Sect. 5.7), feature map spatial importance analysis (Sect. 5.8), and the effects of both data distribution and A-line encoding (Sect. 5.9) are introduced.

### Evaluation metrics

To evaluate the segmentation performance of ACE-Net, region/area and boundary distance-based metrics were computed. Additionally, relevant clinical metrics were determined for the CUBS and IVUS-plaque/calcium datasets for comparison with state-of-the-art methods and for assessing manual segmentation variability. The quantitative experimental results represent the mean and standard deviation obtained on the test set of each dataset.

The Intersection over Union (IoU) (Eq. 5) and the Dice similarity coefficient (DSC) (Eq. 6) were chosen as region metrics to measure the overlap between the GT segmentation and the output of the algorithm’s prediction. For ACE-Net, these metrics were determined using a single contour formed by the union of the upper and lower boundary vectors of the output. The values of IoU and DSC range from 0 to 1, where higher values indicate better segmentation accuracy.

5 $$\begin{aligned} \text {IoU}= &   \frac{|TP|}{|TP| + |FP| + |FN|} \end{aligned}$$

6 $$\begin{aligned} \text {DSC}= &   \frac{2\cdot |TP|}{2\cdot |TP| + |FP| + |FN|} \end{aligned}$$

where TP are the true positives, FP the false positives, and FN the false negatives. TP represents the number of correctly segmented vascular structure pixels, while FP indicates the number of background pixels incorrectly marked as vascular structure. FN represents the number of segmented vascular structure pixels labeled incorrectly as background.

Boundary prediction errors based on distance were assessed using the mean boundary distance (MBD) and the Hausdorff distance (HD). MBD is the average distance between the algorithm-generated boundary coordinates and the GT boundary coordinates. HD is the maximum distance between the algorithm-generated coordinates and the GT coordinates. These metrics were computed over both the upper and lower boundary coordinate vectors of the target vascular structure as follows:

7 $$\begin{aligned} \text {MBD} \!=\! \frac{1}{2K} \left( \sum _{i=1}^{K} d(A_{Ui}, GT_{Ui}) \!+\! \sum _{i=1}^{K} d(A_{Li}, GT_{Li}) \right) \end{aligned}$$

8 $$\begin{aligned} \text {HD}= &   max \, \biggl \{ \hat{H}(A_{U}, GT_{U}), \hat{H}(GT_{U}, A_{U}), \nonumber \\  &   \hat{H}(A_{L}, GT_{L}), \hat{H}(GT_{L}, A_{L}) \biggr \} \end{aligned}$$

where the terms $d(A_{Ui}, GT_{Ui})$ and $d(A_{Li}, GT_{Li})$ represent distances. These distances quantify the spatial separation between the algorithm-generated upper coordinate vector $A_{UB}$ and lower coordinate vector $A_{LB}$, and the corresponding GT upper coordinate vector $GT_{UB}$ and lower coordinate vector $GT_{LB}$ at A-line *i*. Furthermore, $\hat{H}(A_{U}, GT_{U})$ measures the maximum distance between each coordinate $A_{Ui} \in A_{U}$ and its nearest neighbor $GT_{Ui} \in GT_{U}$ along the A-lines. The number of A-lines with a target structure present in the ground truth is denoted by *K*. For A-lines without a target structure, the GT coordinate value is set as *z* (A-line length). This approach introduces a penalty distance boundary error, which effectively addresses false positive detection during the evaluation process.

Different clinical metrics were determined for the CUBS and the IVUS-plaque/calcium datasets due to variations in the imaged vasculature sites. For the CUBS dataset, the carotid intima-media thickness (CIMT) and its bias and absolute bias against the GT values were measured, as described in [34]. Note that computing the CIMT also allows for a direct comparison with the results obtained by the technical study of CUBS [37]. For the IVUS-plaque/calcium dataset, the plaque cross-sectional area (CSA) and calcium arc were determined, which are among commonly used measurements for assessing progression of coronary atherosclerosis [38]. The bias and absolute bias of plaque CSA and calcium arc were used to quantify segmentation performance against GT-generated metrics:

9 $$\begin{aligned} CSA_{Bias}&= CSA_{A} - CSA_{GT} \end{aligned}$$

10 $$\begin{aligned} CSA_{AbsBias}&= |CSA_{A} - CSA_{GT}| \end{aligned}$$

11 $$\begin{aligned} Arc_{Bias}&= Arc_{A} - Arc_{GT} \end{aligned}$$

12 $$\begin{aligned} Arc_{AbsBias}&= |Arc_{A} - Arc_{GT}| \end{aligned}$$

where plaque CSA is determined using the Shoelace algorithm [39] considering its upper and lower boundary vectors to be the vertices of the region polygon. The calcium arc is given by the maximum number of consecutive A-lines with calcium presence. If two separate calcium deposits are present in the same cross-section, only the largest total continuous length of calcium was considered, following the methodology described in [40].

In addition, the *p*-value of the DSC between ACE-Net and state-of-the-art methods on each dataset was computed for statistical analysis using Wilcoxon signed-rank tests. Results were considered statistically significant when *p* < 0.05. Lastly, the inference time to segment one image was determined on the IVUS-plaque/calcium dataset. The average inference time over the length of the dataset was calculated to assess the feasibility of using ACE-Net intra-operatively.

**Fig. 4:**
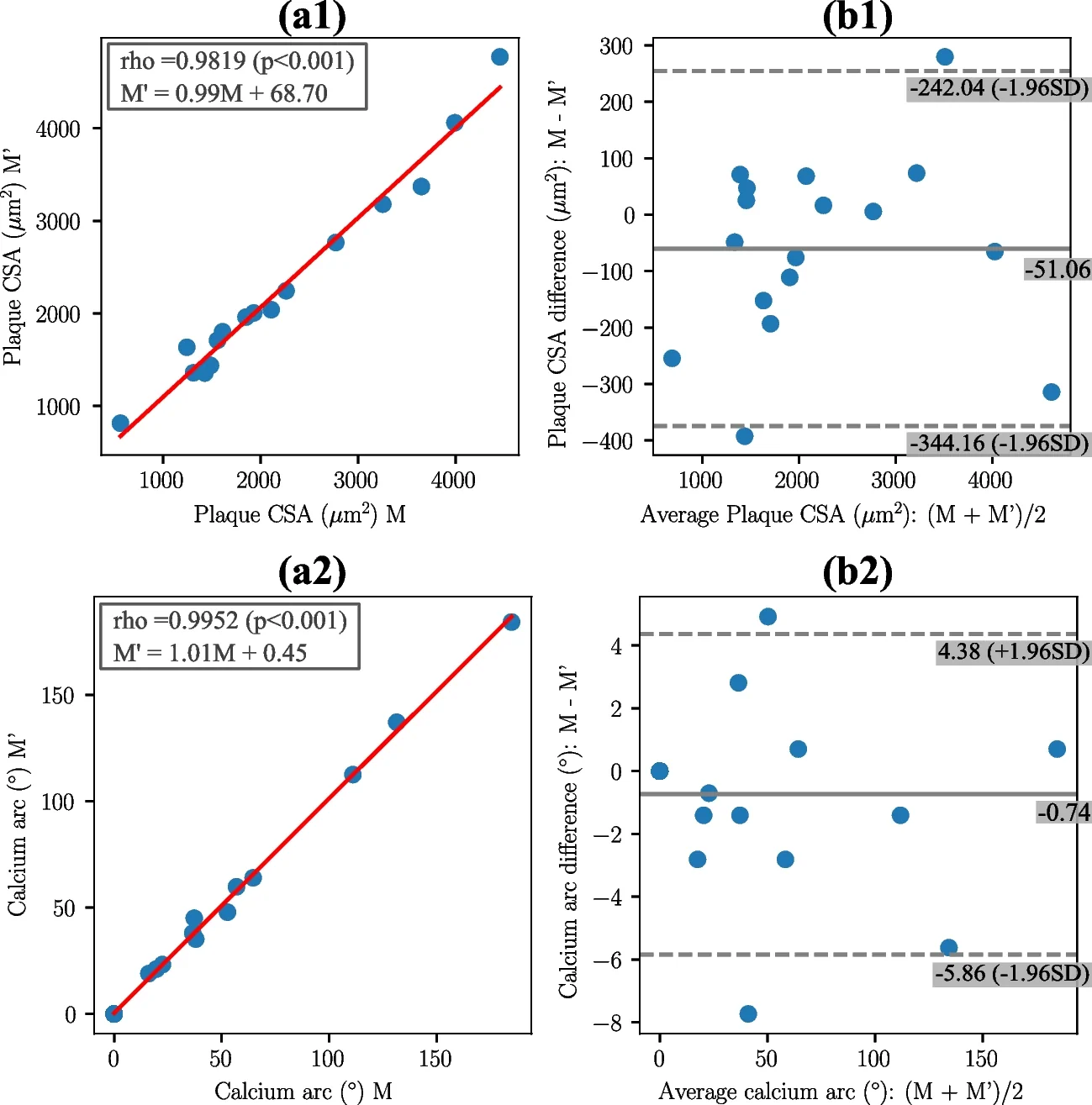
Linear correlation (**a**) and Bland-Altman (**b**) plots of manual plaque CSA (**1**) and calcium arc (**2**) measurements (*n*=20) on two occasions: M and M’. The grey solid line represents the bias (plaque CSA: -51.06 μm$^2$; calcium arc: -0.74 °), and the dotted lines indicate the mean±1.96 SD. (CSA, cross-sectional area)

### Implementation details

For all three datasets, image and label pixel values were normalized and resized, respectively, to [0, 1] and to 256×256. Regarding data splitting for validation, different approaches were taken depending on the dataset. For the CUBS dataset, a random split approach was adopted and 1450 images were used for training and 726 images for testing. The same random split strategy is used for the IVUS-plaque/calcium and IVUS-lumen datasets. In the case of the IVUS-lumen dataset, considering its reduced distribution variability, we adjusted the testing ratio, splitting the images into 500 for training and 500 for testing. Note that both IVUS datasets contain images from all patients in both the training and testing sets. To evaluate the generalization ability of ACE-Net in a more realistic clinical scenario, a three-fold patient-wise cross-validation was also performed on the IVUS-plaque/calcium dataset, where one patient was left out for testing and the remaining patients were used for training. This was repeated three times with different randomly selected test patients.

The proposed ACE-Net is implemented in PyTorch version 1.10.2, with an NVIDIA GeForce RTX 3090 GPU for training. The network was trained using the Adam optimizer with a learning rate of $1\times 10^{-3}$ and $1\times 10^{-4}$ for the early training and fine-tuning stages, respectively. Batch size was set to 2, and training was stopped when the training losses of ACE-Net and state-of-art methods reached a plateau. To prevent over-fitting, we deploy data augmentation on-the-fly for the training of all the networks. For the ACE-Net output, the threshold of *p* was set to 0.6 to binarize negative and positive presence per A-line. Online data augmentation was employed on polar domain images to enlarge the training set (e.g., lateral and axial shifting). In particular, random lateral shifting was used to randomize the reference angle (i.e., 0°) of Cartesian/polar transformation and reduce its impact on network performance. Both the accuracy and speed tests are implemented with a final feature depth $n_x=256$ for ACE-Net. The speed evaluation was carried out with a laptop with a Intel(R) Core(TM) i5-9400 H 2.50GHz CPU and an 8GB GPU NVIDIA QT1000 GPU. On the same laptop, statistical analyses were performed using IBM SPSS Statistics v.27.0 (IBM Corp., Armonk, NY, USA).

**Table 1:** Intra-observer variability analysis for *n*=20 cases at two time points for the IVUS-plaque/calcium dataset

Region	IoU ↑	DSC ↑	MBD (pixel) ↓	HD (pixel) ↓	CSA$_\text {Bias}$ (µm$^2$)	CSA$_\text {AbsBias}$ (µm$^2$)	Arc$_\text {Bias}$ (°)	Arc$_\text {AbsBias}$ (°)
Plaque	0.82±0.08	0.90±0.05	5.25±2.47	21.53±15.98	-51.06±153.42	109.74±116.65	–	–
Calcium	0.82±0.05	0.90±0.03	2.33±1.97	27.07±56.33	–	–	-0.74±2.67	1.65±2.20

Results are presented as mean ± standard deviation. (MBD, mean boundary distance; HD, Hausdorff distance; CSA, cross-sectional area)

**Table 2:** State-of-the-art quantitative comparison results on the CUBS dataset

Method	IoU ↑	DSC ↑	MBD (pixel) ↓	HD (pixel) ↓	CIMT (μm)	CIMT$_{\text {Bias}}$ (μm)	CIMT$_{\text {AbsBias}}$ (μm)
PAN [42]	0.78±0.22	0.86±0.15 $^{\dagger }$	3.95±2.70	15.64±10.34	644±148	-80±154	34±110
FCN [14]	0.74±0.25	0.82±0.18$^{\dagger }$	3.98±2.87	15.82±10.70	1176±198	452±176	454±173
Res-Unet [43]	0.81±0.18	0.88±0.12	3.43±2.15	13.64±7.97	665±133	-58±130	111±90
Unet++ [15]	0.80±0.19	0.87±0.13$^{\dagger }$	3.34±2.23	13.64±8.38	691±162	-29±130	105±82
DeepLabV3 [9]	0.73±0.25	0.82±0.18$^{\dagger }$	3.77±2.45	14.70±9.50	1192±200	468±196	469±194
DeepLabV3+ [44]	0.74±0.25	0.82±0.18$^{\dagger }$	3.51±2.32	**13**.**26**±**8**.**26**	1271±218	548±177	548±176
SETR [10]	0.75±0.24	0.83±0.17$^{\dagger }$	3.80±2.45	14.68±8.21	1011±189	287±198	299±179
CNR$_{\text {IT}}$ [34]	0.65±0.33	0.73±0.27$^{\dagger }$	17.32±14.93	57.45±35.08	707±158	**-17**±**135**	**104**±**87**
TUM$_{\text {DE}}$ [34]	0.76±0.23	0.84±0.17$^\dagger$	8.52±8.59	41.53±29.32	699±163	-24±145	110±96
**ACE-Net**	**0**.**82**±**0**.**18**	**0**.**89**±**0**.**11**	**2**.**84**±**2**.**42**	14.06±8.86	689±179	-36±145	117±93

Results are presented as mean ± standard deviation. Best results are highlighted in *bold*. ↑ and ↓ indicate, respectively, that larger values and smaller values correspond to better performance. The GT CIMT value was of 724±196 µm. In DSC, the dagger (†) defines results statistically significantly different (*p* < 0.05) using the Wilcoxon signed-rank test with respect to ACE-Net. (MBD, mean boundary distance; HD, Hausdorff distance; CIMT, carotid intima-media thickness)

### Manual segmentation variability assessment

In order to provide a reference for algorithm performance, the variability of intra-observer manual segmentation was assessed. In particular, 20 randomly selected images from the IVUS-plaque/calcium dataset were segmented by a trained expert twice with a 6 months interval between segmentations to minimize memory bias [41]. Intra-observer variability was determined by calculating the IoU and DSC for plaque and calcium regions and the distance-based metrics MBD and HD for their respective upper and lower boundary vectors, between the two sets of manual segmentation. In addition, the differences in plaque CSA and calcium arc were computed. Correlation and Bland-Altman analyses were performed on these metrics to better visualize the agreement between the two manual measurements (Fig. 4). The effect of inter-observer variability was not investigated in this work.

Table 1 summarizes the performance of intra-observer manual segmentation. For 20 randomly selected images, plaque and calcium IoUs and DSCs were, respectively, 0.82±0.08 and 0.82±0.05 and 0.90±0.05 and 0.90±0.03. Despite demonstrating similar region overlap accuracy, the MBD was lower for calcium compared to plaque (2.33±1.97 vs 5.25±2.47). This is possibly because the echogenicity of calcium areas contributes to enhanced agreement in manual segmentation. Conversely, the HD was 27.07±56.33 for calcium and 21.53±15.98 for plaque.

### State-of-the-art comparison study

The performance of ACE-Net was first evaluated on the CUBS and IVUS-plaque/calcium datasets by comparing it to recent state-of-the-art methods employed for US segmentation, either based on CNN or transformers approaches: PAN [42], FCN [14], Res-Unet [43], Unet++ [15], DeepLabV3 [9], DeepLabV3+ [44], and SEgmentation TRansformer (SETR) [10]. Although SETR has not been specifically applied to US images in recent works, it is considered as one of the latest state-of-the-art segmentation methods (for generic images). Note that as for these segmentation methods no boundary information is outputted directly, post-processing is carried out to extract ROI boundaries. This is achieved by searching the conjunction locations between positive and negative pixels within each segmentation channel, as described in [45]. For the CUBS dataset, the two best-performing methods from its technical study were also included in this state-of-the-art comparison: CNR$_{\text {IT}}$ and TUM$_{\text {DE}}$ [34]. For the IVUS-plaque/calcium dataset, the A-line-based methods introduced in Sect. 2 were not included in this study as these solely propose A-line classification [22–24].

**Fig. 5:**
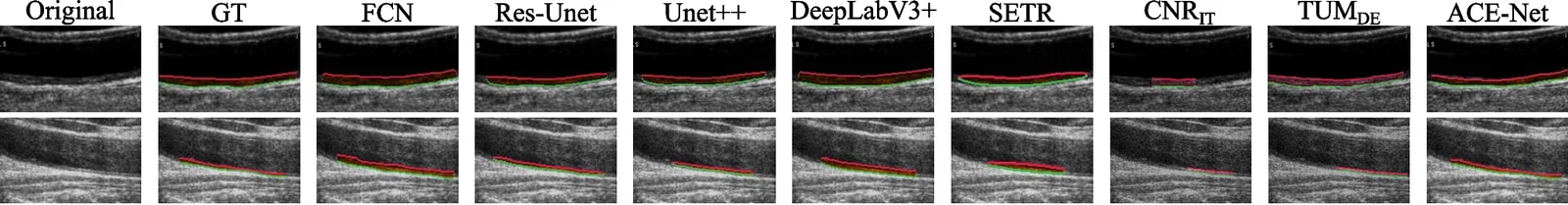
Qualitative comparison of ACE-Net and relevant state-of-the-art methods on the CUBS dataset. The original US image and its GT label are shown. The upper and lower boundaries of the intima-media region are, respectively, delineated in red and green colors. The segmentation boundaries output by both CNR$_\text {IT}$ and TUM$_\text {DE}$ have been interpolated for enhanced qualitative comparison

#### CUBS dataset

The results in Table 2 show that ACE-Net significantly outperforms all state-of-the-art methods considering all segmentation metrics, except for DeepLabV3+ that shows lower, yet similar, accuracy results for HD.

The segmentation result examples in Fig. 5 confirm that while FCN and DeepLabV3+ are able to cover the intima-media area well, their output can be oversized, as shown in the first row of the figure. Moreover, in the case of a thin intima-media layer (2nd row of Fig. 5), unlike ACE-Net, DeepLabV3+, Unet++, and, in particular, SETR tend to misdetect part of the target region.

Furthermore, it can also be seen that Res-Unet, Unet++, and SETR cannot keep the end points of the upper and lower boundaries separated, which contributes to a higher MBD in comparison to ACE-Net (see third column of Table 2).

From the fifth column of Table 2, it can also be observed how the majority of the methods produce similar results in terms of HD, except for CNR$_\text {IT}$ and TUM$_\text {DE}$. This is because CNR$_\text {IT}$ and TUM$_\text {DE}$ present larger missing areas on the left and/or right extremities of the segmented region in comparison to the remaining methods. In the case of CNR$_\text {IT}$, Fig. 5 shows a clear example of the aforementioned missing areas.

Regarding the CIMT, CNR$_\text {IT}$ showed both the lowest bias (-17±135 µm) and absolute bias (104±87 (μm). Nevertheless, ACE-Net, together with Res-Unet, Unet++, and TUM$_\text {DE}$, show comparable CIMT bias and absolute bias to CNR$_\text {IT}$.

**Table 3:** State-of-the-art quantitative comparison on the IVUS-plaque/calcium dataset

Method	Overall		Time (ms) ↓			
	IoU ↑	DSC ↑				
PAN [42]	0.77±0.24	0.84±0.21$^{\dagger }$	16.5			
FCN [14]	0.79±0.24	0.86±0.19$^{\dagger }$	64.7			
Res-Unet [43]	0.82±0.22	0.87±0.20$^{\dagger }$	18.1			
Unet++ [15]	0.87±0.14	0.92±0.10$^{\dagger }$	34.7			
DeepLabV3 [9]	0.79±0.23	0.86±0.19$^{\dagger }$	130.0			
DeepLabV3+ [44]	0.84±0.18	0.90±0.15$^{\dagger }$	17.8			
SETR [10]	**0**.**88**±**0**.**12**	**0**.**93**±**0**.**09**$^{\dagger }$	30.3			
**ACE-Net**	0.87±0.12	**0**.**93**±**0**.**09**	**12**.**1**$^{*}$			
Method	Plaque					
	IoU ↑	DSC ↑	MBD (pixel) ↓	HD (pixel) ↓	CSA$_\text {Bias}$ ((μm$^2$)	CSA$_\text {AbsBias}$ ((μm$^2$)
PAN [42]	0.69±0.21	0.79±0.22$^{\dagger }$	7.30±6.38	29.56±27.23	476±397	454±292
FCN [14]	0.76±0.22	0.85±0.10$^{\dagger }$	7.47±6.80	32.64±30.06	235±252	240±172
Res-Unet [43]	0.73±0.27	0.80±0.30$^{\dagger }$	5.17±5.41	29.37±29.87	161±267	196±194
Unet++ [15]	**0**.**84**±**0**.**10**	**0**.**91**±**0**.**09**	3.91±2.94	21.60±24.72	**110**±**248**	179±194
DeepLabV3 [9]	0.75±0.16	0.85±0.15$^{\dagger }$	6.94±5.67	32.88±29.26	251±243	247±161
DeepLabV3+ [44]	0.81±0.14	0.89±0.13	4.35±2.62	23.82±21.74	185±246	208±165
SETR [10]	**0**.**84**±**0**.**08**	**0**.**91**±**0**.**05**$^{\dagger }$	3.46±2.45	12.13±13.66	155±251	197±184
**ACE-Net**	**0**.**84**±**0**.**08**	**0**.**91**±**0**.**05**	**2**.**65**±**1**.**66**	**8**.**71**±**5**.**32**	120±136	**115**±**101**
Method	Calcium					
	IoU ↑	DSC ↑	MBD (pixel) ↓	HD (pixel) ↓	Arc$_\text {Bias}$ (°)	Arc$_\text {AbsBias}$ (°)
PAN [42]	0.53±0.28	0.66±0.25$^{\dagger }$	3.56±4.72	22.27±30.14	15±40	19±38
FCN [14]	0.50±0.28	0.62±0.29$^{\dagger }$	3.97±5.41	21.19±30.39	**3**±**50**	38±50
Res-Unet [43]	0.68±0.20	0.79±0.15$^{\dagger }$	2.19±3.25	15.54±26.74	7±11	9±10
Unet++ [15]	0.69±0.18	0.81±0.13$^{\dagger }$	1.85±1.81	9.05±18.81	**3**±**14**	9±14
DeepLabV3 [9]	0.49±0.30	0.63±0.26$^{\dagger }$	3.73±5.13	17.73±27.60	**3**±**47**	36±47
DeepLabV3+ [44]	0.62±0.24	0.74±0.20$^{\dagger }$	2.28±2.62	11.21±21.74	7±10	9±9
SETR [10]	**0**.**75**±**0**.**16**	**0**.**85**±**0**.**13**$^{\dagger }$	1.88±4.26	**4.95±11.21**	9±17	7±17
**ACE-Net**	0.73±0.16	0.84±0.12	**1**.**69**±**1**.**23**	9.10±11.63	**3**±**4**	**5**±**4**

The overall scores consider healthy tissue, plaque, and calcium areas. Results are presented as mean ± standard deviation. Best results are highlighted in *bold*. ↑ and ↓ indicate, respectively, that larger values and smaller values correspond to better performance. In DSC, the dagger (†) defines results statistically significantly different (*p* < 0.05) using the Wilcoxon signed-rank test with respect to ACE-Net. Note that the end-to-end computation time of ACE-Net (*) includes conversions between Cartesian and polar domains twice, while for other segmentation methods, only network forward inference time is included (MBD, mean boundary distance; HD, Hausdorff distance; CSA, cross-sectional area)

**Fig. 6:**
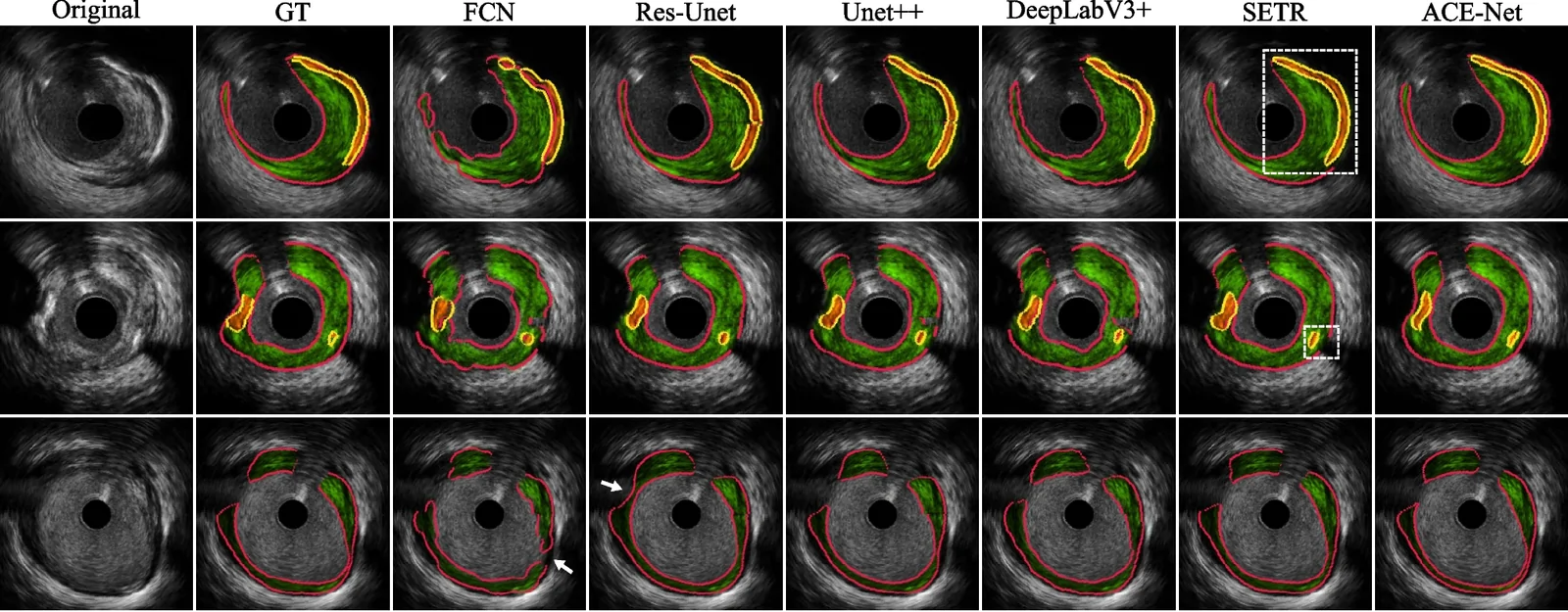
Qualitative comparison of ACE-Net and relevant state-of-the-art methods with 3 representative cases (1st and 2nd rows: plaque and calcium; 3rd row: only plaque). The original IVUS image and its GT label are shown. Green mask with red contour indicates areas of plaques. Orange masks with yellow contour indicate areas of calcium. Note that the upper and lower boundaries output by ACE-Net were merged in a single boundary for better comparison. Examples of plaque mask segmentation errors and false discontinuous contours are, respectively, highlighted in FCN and Res-Unet (arrows) and in SETR (dashed boxes)

#### IVUS-plaque/calcium dataset

Table 3 reports the performance of ACE-Net relative to state-of-the-art methods on the IVUS-plaque/calcium dataset with random data splitting. Note that CNR$_\text {IT}$ and TUM$_\text {DE}$ are not included in this assessment because their results on the CUBS dataset (Sect. 5.4.1) were obtained from the dataset’s public repository. Figure 6 presents the corresponding qualitative results (boundaries and/or masks). More examples are available in the supplementary materials.

Across region-based metrics, ACE-Net shows similar accuracy to SETR and Unet++, in particular for overall and plaque areas. Regarding calcium areas, the transformers-based method SETR achieves the highest overall region accuracy (DSC of 0.85±0.13), with only a 1% difference from ACE-Net (DSC of 0.84±0.12). Statistically significant differences (*p* < 0.05) were found between ACE-Net and all state-of-the-art segmentation methods, except in the case of Unet++ and DeepLabV3+ for plaque regions.

For distance-based metrics, ACE-Net outperforms all state-of-the-art methods for MBD for both plaque and calcium. In terms of HD, ACE-Net outperforms all other methods for plaque segmentation, while SETR achieves the lowest HD value for calcified areas. Vascular structures present around the left and right edges of an input image may contribute to an increase of HD values for ACE-Net.

It can be further verified in Fig. 6 that even though SETR achieved good plaque segmentation from its output pixel masks, the post-processed boundary extraction resulted in fragmented contours due to missing pixels in the predicted mask. Conversely, ACE-Net directly outputs clear segmentation boundary vectors (and post-processed pixel masks) of target regions in an end-to-end fashion.

The clinical metrics comparison showed that all evaluated methods exhibited a tendency to overestimate the values of plaque CSA. Among the assessed techniques, Unet++ demonstrated the best performance in accurately estimating CSA (110±0.13), followed closely by ACE-Net (120±0.13). Similarly, in terms of estimating calcium arc, all methods displayed a tendency to overestimate its values. Notably, ACE-Net, along with FCN, Unet++, and DeepLabV3, outperformed the remaining approaches in accurately estimating the arc. Specifically, ACE-Net achieved calcium arc bias and absolute bias values of 3±4 and 5±4, respectively. These results indicate a slight positive deviation from the intra-observer variability analysis results (Sect. 5.3), which can be taken as reference values.

Moreover, an end-to-end contour segmentation using ACE-Net can process more than 82 frames per second (FPS) on a laptop computer (faster than the considered state-of-the-art methods), which meets the requirements of a real-time intra-operative application. If ACE-Net is only used for quantitative analysis and Cartesian masks are not needed for display, a speed of 123 FPS (8.1 ms per frame, 2×-10× faster than state-of-the-art methods) can be achieved on the same hardware setting.

### Ablation study

**Fig. 7:**
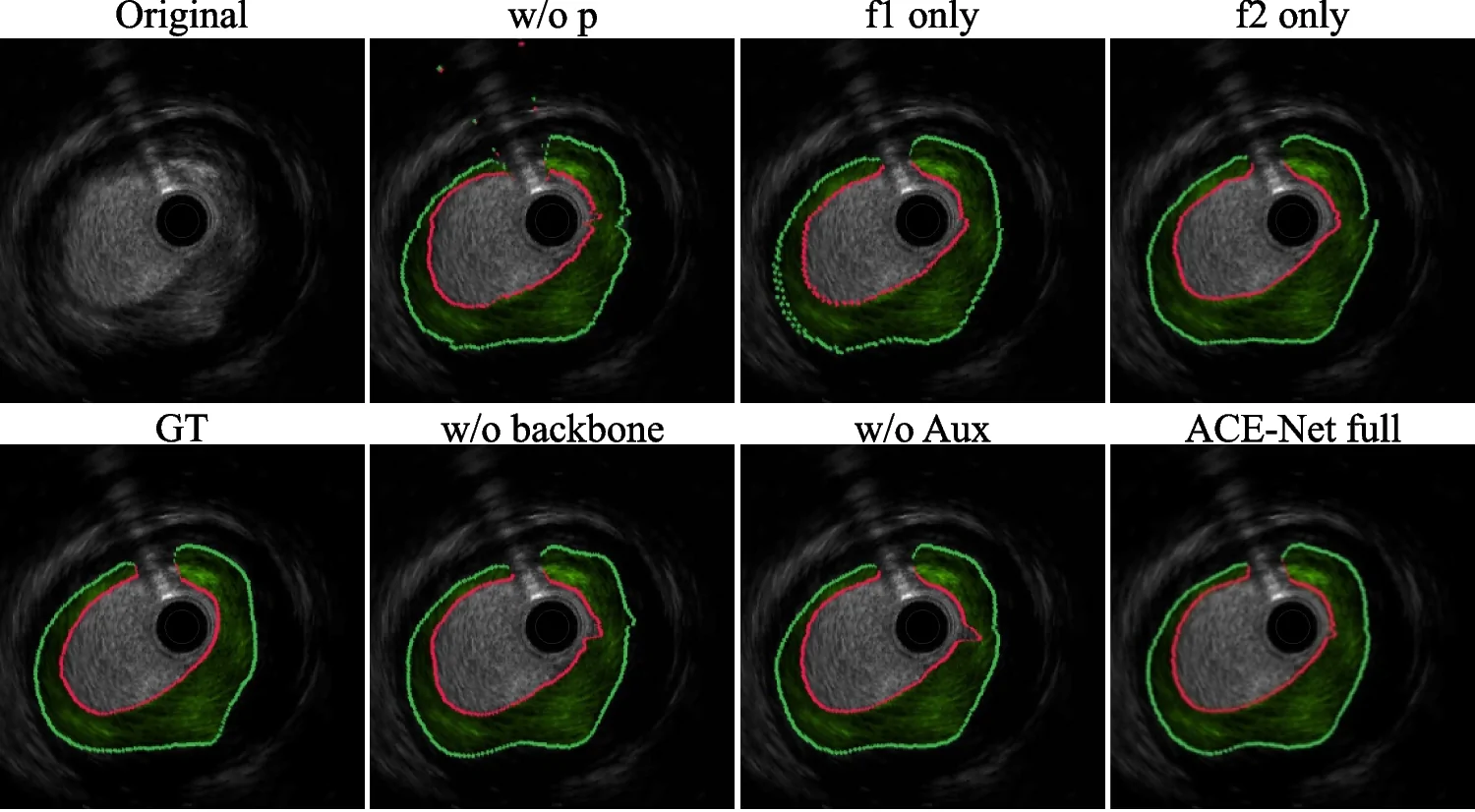
Example of the ablation experiment qualitative results on the IVUS-plaque/calcium dataset. Green masks indicate areas of plaque. Upper and lower boundaries of plaque are delineated in red and green, respectively (w/o, without)

An ablation study was carried out on the IVUS-plaque/calcium dataset by removing the following parts of ACE-Net: A-line feature branches $f_i$, backbone module, auxiliary encoder, and presence probability *p* encoder. The obtained quantitative results are summarized in Table 4. A qualitative sample of the ablation study is shown in Fig. 7.

**Fig. 8:**
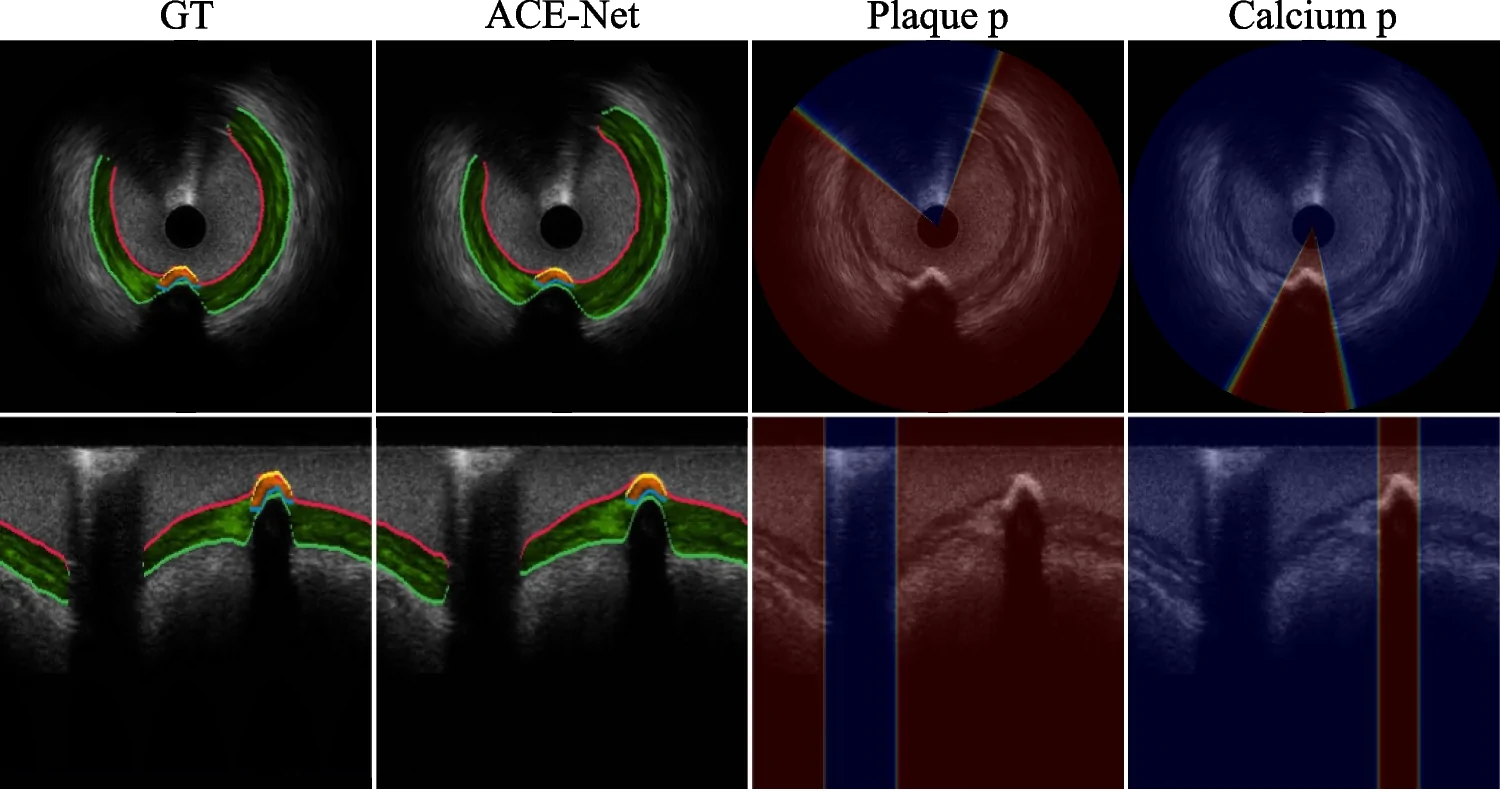
ACE-Net output Cartesian (1st row) and polar (2nd row) domain representations of a case from the IVUS-plaque/calcium dataset. GT contours are depicted on the first column. ACE-Net predicted boundaries and presence probability masks (for all A-lines) of plaque and calcium are shown in the last three columns. Green and orange masks indicate areas of plaque and calcium. Upper and lower boundaries of plaque and calcium are delineated in red, green, yellow, and blue, respectively

Results indicate that the aforementioned ACE-Net components contribute to the accuracy of the network, with the presence probability *p* encoder removal primarily impacting its robustness. Without *p*, ACE-Net predicts wrong coordinates at the conjunction A-lines between presence and non-presence areas, and because of average of loss between these areas, the estimation accuracy on presence areas is degraded. *p* is thus essential for regressing clean discontinuous coordinate vectors since, by predicting *p*, a clear AOI mask with sharp edges can be produced (see Fig. 8).

**Table 4:** Ablation study on different ACE-Net components

Setup	Overall		Plaque				Calcium			
	IoU ↑	DSC ↑	IoU ↑	DSC ↑	MBD (pixel) ↓	HD (pixel) ↓	IoU ↑	DSC ↑	MBD (pixel) ↓	HD (pixel) ↓
w/o *p*	0.73±0.26	0.81±0.22$^{\dagger }$	0.70±0.18	0.81±0.16$^{\dagger }$	4.73±3.48	67.71±41.69	0.35±0.34	0.50±0.28$^{\dagger }$	6.48±5.05	88.44±52.58
$f_1$ only	0.79±0.22	0.86±0.17$^{\dagger }$	0.77±0.17	0.86±0.16$^{\dagger }$	3.46±2.91	17.65±21.56	0.50±0.28	0.65±0.22$^{\dagger }$	4.35±4.12	21.02±19.95
$f_2$ only	0.81±0.19	0.88±0.15$^{\dagger }$	0.77±0.16	0.86±0.15$^{\dagger }$	3.12±2.38	16.03±19.66	0.57±0.24	0.71±0.17$^{\dagger }$	2.41±1.82	16.59±17.82
w/o backbone	0.83±0.20	0.89±0.18$^{\dagger }$	0.75±0.23	0.82±0.24$^{\dagger }$	3.44±3.98	45.76±33.40	0.69±0.21	0.80±0.16$^{\dagger }$	1.90±2.52	20.29±23.02
w/o Aux	0.83±0.18	0.89±0.14$^{\dagger }$	0.79±0.12	0.88±0.09$^{\dagger }$	3.53±2.52	14.69±15.49	0.61±24	0.74±0.19$^{\dagger }$	2.44±1.92	12.59±12.86
**ACE-Net full**	**0**.**87**±**0**.**12**	**0**.**93**±**0**.**08**	**0**.**83**±**0**.**08**	**0**.**9**1±**0**.**05**	**2**.**65**±**1**.**66**	**8**.**71**±**5**.**32**	**0**.**73**±**0**.**16**	**0**.**84**±**0**.**12**	**1**.**69**±**1**.**23**	**9**.**10**±**11**.**63**

The overall scores consider healthy tissue, plaque, and calcium areas. Results are presented as mean ± standard deviation. Best results are highlighted in *bold*. ↑ and ↓ indicate, respectively, that larger values and smaller values correspond to better performance. In DSC, the dagger (†) defines results statistically significantly different (*p* < 0.05) using the Wilcoxon signed-rank test with respect to ACE-Net full. (w/o, without; MBD, mean boundary distance; HD, Hausdorff distance)

**Fig. 9:**
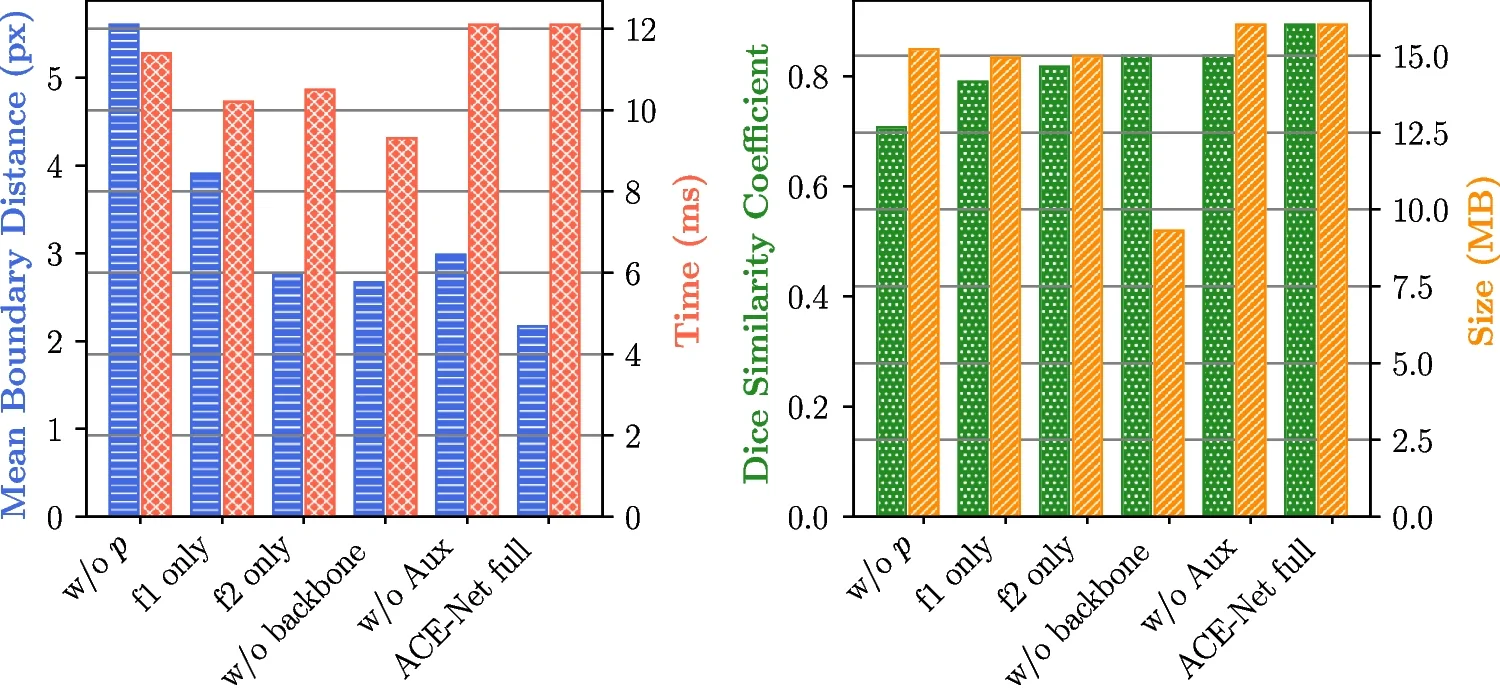
Accuracy/speed trade-off of the ablation study for the proposed ACE-Net on the IVUS-plaque/calcium dataset. The inference time vs the MBD error and the network size vs the DSC are shown

When using a single branch of the A-line feature extractor and removing other parallel branches, the overall accuracy is lower than a full multi-branch architecture. The dense branch $f_1$ regresses contour coordinates focusing more on local A-lines, generating a noisy predicted boundary. When only using branch $f_2$, the obtained coordinate vector is smoother, but the vector will underfit complex shapes. These findings suggest that a multi-scale A-line coordinate encoding is a highly accurate and efficient way of estimating coordinate vectors in US images.

Additionally, it can be noted that ACE-Net can still achieve considerable accuracy in comparison with other state-of-the-art methods (see Sect. 5.4.2) without the backbone module, whereby images are directly fed to the A-line feature extractor.

Lastly, even if having the least impact on the accuracy of the network, adding an auxiliary encoder to the training of ACE-Net further improves its accuracy as the A-line feature branches are forced to perform in a similar fashion.

The comparison between ACE-Net full and its ablated variants revealed statistically significant differences in the obtained DSC values, supporting the consistent superiority of the proposed method. This superiority is further evidenced by the remaining evaluated metrics.

Figure 9 reports the accuracy/speed trade-off of the ablation study. Results indicate that adding auxiliary loss introduces zero burden to the computation time and memory size while increasing the boundary accuracy. Also, even though removing the backbone resulted in a reduction of 7.9% of the DSC in comparison with the full ACE-Net, it showed 35.4% less inference time (decrease from 7.9 to 5.1 ms) and 41.7% less memory (decrease from 16.1 to 9.3 MB). These results suggest that ACE-Net does not require a backbone to achieve comparable accuracy results.

**Table 5:** Effect of training strategy on ACE-Net performance

# training strategy	IoU ↑	DSC ↑	MBD (pixel) ↓
1 – W/o $\mathcal {L}_I$	0.79±0.14	0.86±0.11$^{\dagger }$	3.35±3.09
2 – W/o frozen backbone	0.76±0.14	0.84±0.12$^{\dagger }$	4.48±4.71
3 – ACE-Net One stage	0.80±0.12	0.88±0.09	3.26±2.41
4 – **ACE-Net full**	**0**.**87**±**0**.**12**	**0**.**93**±**0**.**08**	**2**.**65**±**1**.**66**

Results are presented as mean ± standard deviation. Best results are highlighted in *bold*, and ↑ and ↓ indicate, respectively, that larger values and smaller values correspond to better performance. In DSC, the dagger (†) defines results statistically significantly different (*p* < 0.05) using the Wilcoxon signed-rank test with respect to ACE-Net full. (W/o, without; MBD, mean boundary distance; HD, Hausdorff distance)

**Table 6:** Unseen patients evaluation on the IVUS-plaque/calcium dataset

Method	Patient-A			Patient-B			Patient-C		
	IoU ↑	DSC ↑	MBD (pixel) ↓	IoU ↑	DSC ↑	MBD (pixel) ↓	IoU ↑	DSC ↑	MBD (pixel) ↓
Baseline [43]	0.65±0.17	0.71±0.16	**13**.**89**±**14**.**23**	0.63±0.15	0.73±0.14	15.53±10.5	0.60±0.16	0.67±0.17$^{\dagger }$	37.83±33.19
ACE-Net one stage	0.61±0.18	0.68±0.17$^{\dagger }$	17.7±10.32	0.59±0.17	0.67±0.17$^{\dagger }$	18.35±12.54	0.60±0.17	0.69±0.15$^{\dagger }$	26.07±25.12
**ACE-Net full**	**0**.**71**±**0**.**12**	**0**.**81**±**0**.**56**	16.7±12.59	**0**.**64**±**0**.**12**	**0**.**74**±**0**.**11**	**14**.**43**±**10**.**05**	**0**.**62**±**0**.**18**	**0**.**69**±**0**.**17**	**22**.**98**±**19**.**05**

Results are presented as mean ± standard deviation. Best results are highlighted in *bold*, and ↑ and ↓ indicate, respectively, that larger values and smaller values correspond to better performance. In DSC, the dagger (†) defines results statistically significantly different (*p* < 0.05) using the Wilcoxon signed-rank test with respect to ACE-Net full. (MBD, mean boundary distance; HD, Hausdorff distance)

### Effect of multi-task learning and freezing strategy

To assess the impact of the training strategy on the performance of ACE-Net, the proposed two-stage training strategy was compared with three other modified strategies on the IVUS-plaque/calcium dataset. First, the pixel loss $\mathcal {L}_I$ was disabled, and the networks were optimized with only $\mathcal {L}_c$ and $\mathcal {L}_p$ during the training process. Second, we used $\mathcal {L}_I$ to optimize the backbone module, and when back propagating in $\mathcal {L}_c$ and $\mathcal {L}_p$, the backbone was not frozen. Third, we froze the backbone when using $\mathcal {L}_c$ and $\mathcal {L}_p$ throughout training, resulting in only using coordinates and presence losses to optimize the bottom module. We refer to this last strategy as “one stage.”

As shown in Table 5, the second strategy had the worst performance since, most likely, the gradient from $\mathcal {L}_c$ and $\mathcal {L}_p$ conflicts with the gradient from $\mathcal {L}_I$. Also, simply using the coordinate and presence losses (first strategy) showed better results than purely mixing all the losses (second strategy). The pixel loss is useful when the backbone optimization is isolated from the optimization of coordinate/presence encoding (third strategy). No statistically significant difference (*p*≥0.05) was found between the proposed and the third strategies. Nonetheless, by adding a fine-tuning stage, as in the proposed training strategy, ACE-Net performance was further improved.

### Unseen patient evaluation on the IVUS-plaque/calcium dataset

The previous benchmark comparison of the IVUS-plaque/calcium dataset with state-of-the-art methods (Sect. 5.4.2) disregarded the impact of data leakage. However, when using deep learning for medical image analysis, a new patient who was not included in the previous training will usually be encountered. To assess this, as described in Sect. 5.2, we performed a three-fold patient-wise cross-validation on the IVUS-plaque/calcium dataset. In this experiment, Res-Unet [43] was used as the baseline. We also compared it to the one-stage training approach described in Sect. 5.6. The results are presented in Table 6.

The overall accuracy of ACE-Net on unseen patients was lower than the results previously reported in Table 3 (e.g., the IoU decreased from 0.8 to 0.65). This discrepancy is likely due to differences between patients in plaque and calcium characteristics, such as density or homogeneity. The testing images may thus include patterns not present in the training set, leading to a distribution mismatch and lower performance. Nevertheless, ACE-Net was still higher than the baseline model, which had an IoU of around 0.62.

The proposed two stages training strategy is crucial for supporting the generalization of ACE-Net, since the one-stage training (i.e., with frozen backbone) had a worse overall performance than the baseline, achieving only an average IoU of 0.60. Statistically significant differences (*p* < 0.05) were found for all three patients when comparing a one-stage ACE-Net with ACE-Net full. Additionally, patient-C exhibited a statistically significant difference for the baseline model compared to ACE-Net full.

**Fig. 10:**
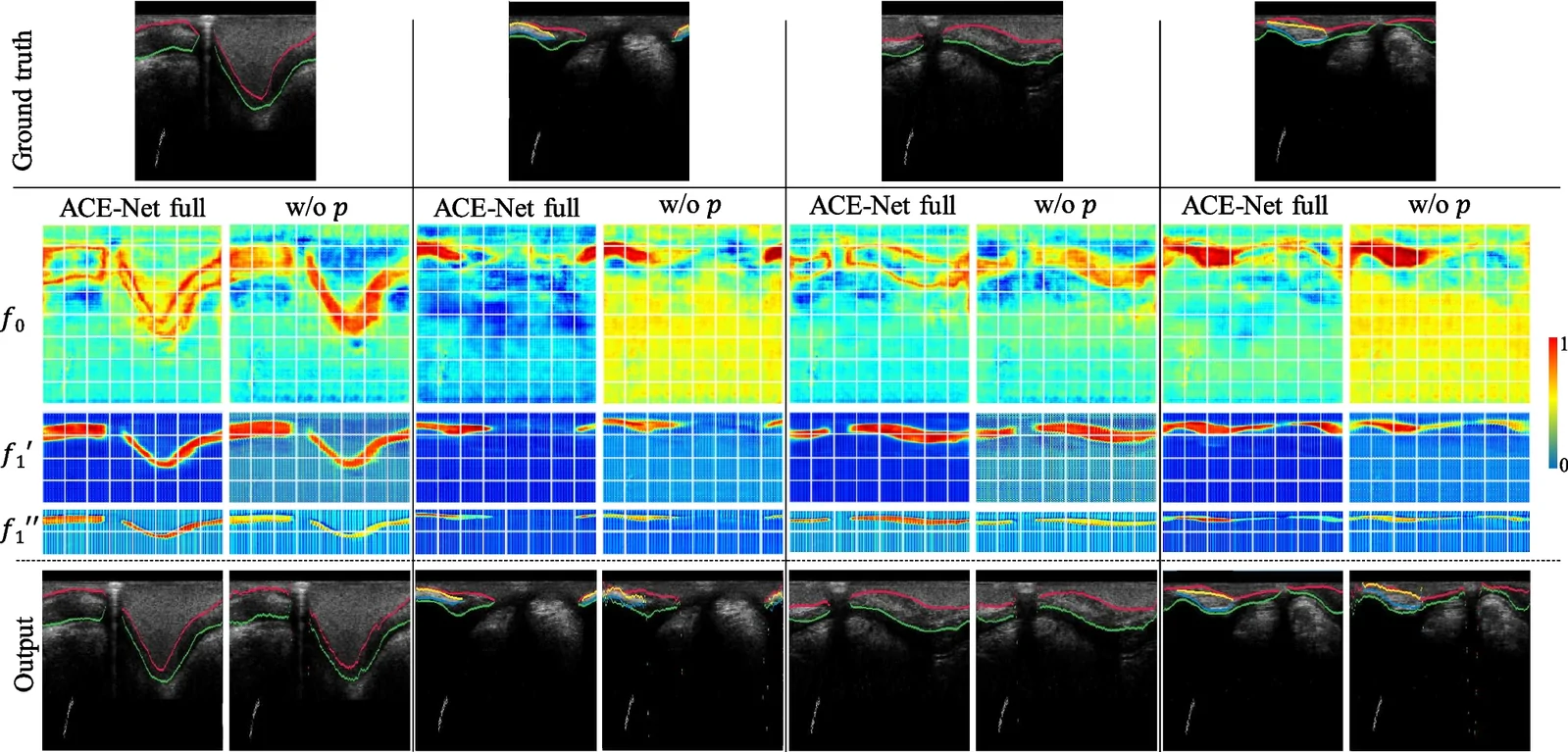
Visualization of feature maps in ACE-Net. The results for both the full ACE-Net and its variant without *p* encoding are shown. $f_0$ is the backbone feature map, and $f_1'$ and $f_1''$ are intermediate feature maps corresponding to the first and second downsampling stages in branch $f_1$, respectively. The feature maps are visualized by projecting them along the depth dimension and normalizing the projected heatmaps. The ground truth and output images depict the contour of plaque and calcium with divided upper and lower boundaries

### Feature map spatial importance analysis

The design of the ACE-Net architecture and its training strategy prioritize the spatial correspondence of low-level features. Inspired by works that analyze the spatial attention of input images, such as the encoding schemes in transformers and visual transformers [25, 26], we investigate which part of a feature map the network is focusing on to predict the location of the boundary. The spatial importance of three low-level feature maps is analyzed: the backbone feature $f_0 \in {{\mathbb {R}}^{x \times z \times n_0 }}$, and two intermediate feature maps $f_1' \in {{\mathbb {R}}^{x \times z/2 \times n_1 }}$ and $f_1'' \in {{\mathbb {R}}^{x \times z/4 \times n_2 }}$, corresponding to the first and second downsampling stages in the A-line encoding branch $f_1$, respectively. The spatial importance of the feature map is visualized by projecting the feature maps along the depth dimension using the absolute value average. For example, $f_0$ is projected to $F_0\in {{\mathbb {R}}^{x \times z}}$ for display, while $f_1'$ and $f_1''$ are also projected to maintain their axial and lateral dimensions instead of the input image dimension. Considering the ablation study described in Sect. 5.5, Fig. 10 presents the results of full ACE-Net and its variant without *p*. For both conditions, the results indicate that the low-level features are focused on pathological areas with higher activation values. As shown in the second row of the figure, compared to the variant without *p* encoding, the $f_0$ feature of the full ACE-Net is more focused on the contour area of the object instead of the whole object. Additionally, for the full ACE-Net, the two higher-level features $f_1'$ and $f_1''$ generally have higher activation in pathological areas, compared to when the *p* encoding is removed. These observations suggest that the full ACE-Net features have better spatial correspondence and attend to feature locations that contribute to the higher accuracy of the boundary coordinates regression.

### Effects of data distribution and A-line encoding

The effects of data distribution and of the proposed A-line coordinates encoding strategy in the overall segmentation accuracy of ACE-Net were evaluated by investigating the contribution of CE (see Fig. 2) and the impact of widening the training contour coordinate distribution with synthetically generated data. Widening the training contour coordinate distribution consisted of increasing the variance of the location of the coordinates within each image. Two different backbones were used in this evaluation: a shallower backbone with 25 output features ($f_0$-25-S) and a deeper backbone with 500 output features ($f_0$-500-L). All experiments were carried out using the IVUS-lumen dataset, described in Sect. 4.3. Note that for the other experiments carried out in this paper, $f_0$-500-L was the backbone used. $f_0$-25-S was here used to better highlight that, even for a shallower backbone, which allows ACE-Net to be even faster, the proposed A-line coordinates encoding allows ACE-Net to reach the baseline accuracy. DeepLabV3+ [44] was set as the baseline because it was the second fastest approach in the state-of-the-art comparison (Table 3).

**Table 7:** Evaluation of the effect of data distribution and A-line encoding on ACE-Net using the IVUS-lumen dataset alone and mixed with synthetically generated data for training

Method	Original IVUS-lumen		Mixed with synthetic	
	IoU ↑	MBD (pixel) ↓	IoU ↑	MBD (pixel) ↓
Baseline [44]	0.93±0.02	6.46±1.99	0.93±0.02	5.63±1.92
$f_0$-25-S w/o CE$^{*}$	0.87±0.04$^{\dagger }$	12.78±4.79	0.87±0.04	12.90±4.51
$f_0$-25-S ACE-Net full	0.91±0.03$^{\dagger }$	4.17±1.76	0.93±0.02	4.06±1.64
$f_0$-500-L w/o CE$^{*}$	**0**.**94**±**0**.**02**	5.90±1.91	0.93±0.02	6.07±2.12
$f_0$-500-L ACE-Net full	0.93±0.02	**3**.**95**±**1**.**84**	**0**.**94**±**0**.**02**	**3**.**71**±**1**.**65**

Results are presented as mean ± standard deviation. Best results are highlighted in *bold*, and ↑ and ↓ indicate, respectively, that larger values and smaller values correspond to better performance. In IoU, the dagger (†) defines results statistically significantly different (*p* < 0.05) using the Wilcoxon signed-rank test with respect to the baseline. Note that without CE (*), coordinates are extracted from IPE using the approach described in [45]. (w/o, without; CE, coordinates encoding; MBD, mean boundary distance)

A total of 5000 synthetic images and upper/lower boundary sets of the lumen region were generated by applying a tissue geometry warping algorithm to this dataset (further details are available in ACE-Net’s public repository). The warping algorithm used in this work extends the horizontal warping approach described in [46]. These synthetic images were mixed with the training set of the original IVUS-lumen dataset to enrich the contour coordinate distribution. The effect of data distribution on ACE-Net is thus assessed by training ACE-Net using either the original or the mixed training sets and then evaluating it on the same testing set, containing only original images. To analyze its contribution, CE was removed from ACE-Net and lumen boundaries were extracted from the pixel-wise semantic segmentation map $I_{PE}$, using the approach described in [45].

The results of the aforementioned assessment are summarized in Table 7. Results indicate that the segmentation accuracy of ACE-Net decreases without CE, in particular for a small backbone (i.e., $f_0$-25-S), which has shown to underfit for both training sets. On the other hand, when using CE and a mixed training set, the performance of ACE-Net improves; ergo, a wider distribution of boundary coordinates, even if by means of synthetic data, is beneficial for the learning of CE. It can be noted that $f_0$-25-S obtains comparable accuracy to $f_0$-500-S, particularly for the mixed training set. For the segmentation of simpler shapes of intravascular structures, e.g., the lumen region, a small backbone could thus be sufficient to achieve satisfactory accuracy. Finally, the statistical analysis conducted with respect to the baseline revealed statistically significant differences (*p* < 0.05) solely for $f_0$-25-S both without CE and ACE-Net full.

## Discussion and conclusions

This paper presents ACE-Net, a novel encoding method and efficient network architecture for vascular structure segmentation in US images. This novel A-line-based end-to-end deep network was evaluated on different segmentation tasks from three US datasets: CUBS, IVUS-plaque/calcium, and IVUS-lumen (Sect. 4). The extensive evaluation conducted on the proposed method demonstrated that by encoding ROIs upper and lower boundaries in A-line coordinates, ACE-Net efficiently predicts clean segmentation masks and further directly extracts highly abstract coordinate information at a fast speed (8-12.1 ms for a frame with a laptop GPU) in different types of US images. ACE-Net also provides direct indications of vascular structures through the utilization of a presence vector-classification mechanism. From the output of ACE-Net, relative distances between segmented structures are easily obtained in real time, enabling the intra-operative computation of clinical metrics.

ACE-Net achieved overall superior performance in accuracy and speed compared to relevant state-of-the-art methods (e.g., Unet, DeepLab, SETR) on the CUBS, and IVUS-plaque/calcium datasets (Sect. 5.4). Considering the recent strong interest in transformer-based models for image segmentation, the comparison between ACE-Net and SETR is particularly interesting. When comparing these two methods on the IVUS-plaque/calcium dataset, the region-based metrics (IoU and DSC) showed similar accuracy. On the CUBS dataset, ACE-Net outperformed SETR in terms of accuracy. This discrepancy can be attributed to the encoding scheme utilized in ACE-Net. The A-line contour representation employed in ACE-Net effectively handles thin strip-shaped objects encountered in the CUBS dataset, while SETR patch-based approach faces challenges with such shapes. Additionally, it is important to note that ACE-Net outputs coordinate vectors, whereas SETR outputs pixel masks. Therefore, for computing metrics like IoU and DSC, pixel masks had to be post-processed from the ACE-Net output. Despite this additional step, the performance of ACE-Net remained comparable to SETR on the IVUS-plaque/calcium dataset, highlighting the robustness of our proposed approach. Furthermore, ACE-Net outperformed SETR in terms of MBD, further emphasizing the advantage of the proposed approach based on coordinate vectors.

In addition to its improved accuracy, ACE-Net exhibited notable efficiency gains in terms of speed. The computational time required for ACE-Net was significantly shorter compared to the other approaches, making it highly suitable for intra-operative clinical applications. The combination of improved accuracy and computational efficiency positions ACE-Net as a promising segmentation solution during US-guided procedures. For example, in the context of PCI, the utilization of ACE-Net could potentially streamline clinical workflow and planning and reduce operator dependency when assessing atherosclerotic lesions and markers.

ACE-Net could reduce operator dependency by supporting the direct computation of clinical metrics from its output vectors. These metrics help diagnose and monitor vascular conditions based on thickness, area, arc, and other parameters. Moreover, ACE-Net can generate pseudo boundary labels for fast auto-annotation of target ROIs, unlike semantic segmentation methods.

The fundamental role of *p* encoding for the accuracy of ACE-Net was determined with an ablation study (see Sect. 5.5) and analyzed in detail with the feature visualization described in Sect. 5.8. The findings described in the aforementioned sections revealed that when utilizing *p* encoding, the feature extraction module is focused on the contour aspect of ROIs. This is particularly relevant in the presence of image artifacts that cause true contour discontinuities, such as a guidewire shadow in IVUS images. It was noted that satisfactory accuracy (Table 4) and efficiency (Fig. 9) results can be achieved even without a backbone.

The impact of data leakage was assessed on the IVUS-plaque/calcium dataset (Sect. 5.7). A patient-wise splitting approach was employed, and ACE-Net was compared to its one-stage variant and a baseline network (Res-Unet). The results confirmed that our comprehensive training strategy improved ACE-Net generalization. However, the performance of this experiment was lower than that of random splitting. This result not only indicates a higher level of distribution shift in the patient-wise splitting experiment but also suggests that the dataset lacked sufficient diversity and representativeness for individual patients. Expanding the dataset to include more patients and a wider range of conditions could enhance ACE-Net’s accuracy for unseen patient data.

Finally, ACE-Net performance was shown to improve by training with synthetic data (Sect. 5.9). Furthermore, by using an MTL strategy to help the backbone module learn dense spatial features, the coordinate regression accuracy of ACE-Net increased significantly compared to a training strategy with only coordinates and presence probability losses (Sect. 5.6). Overall, the experiments carried out demonstrated the positive impact of ACE-Net’s modules and our two-stage training pipeline, all designed for the proposed A-line-based encoding scheme.

### Limitations

Cartesian representations are typically favored in a clinical setting for the visualization of, e.g., IVUS images. However, due to the proposed A-line encoding scheme, a limitation of ACE-Net is the need of input images in polar domain, which might create a dependence on the reference 0$^\circ$ angle and/or cause a contour discontinuity at the same angle between the Cartesian-polar conversion of image.

During the training phase, we used horizontal shifting to reduce the potential dependence on the reference 0$^\circ$ angle between the Cartesian-polar conversion of images. This approach enhances the versatility and reliability of ACE-Net in practical scenarios, by improving its robustness and generalization capabilities.

Nonetheless, since the first and last A-lines of polar domain images are not conjoined, a predicted contour coordinates vector could still present a discontinuity at the 0$^\circ$ A-line (or equivalently 360$^\circ$) when converted to its Cartesian domain. This may occur when a polar image happens to have weak contour features at the left border or at the right border. Yet, this could be addressed in two ways. First, even if requiring additional processing for the cases with no contour at the image border, the use of a continuity loss term in training [46] or post-filtering of the predicted coordinates vector would forcibly connect the 0$^\circ$ and 360$^\circ$ A-line contour points in the Cartesian domain. Second, the original polar domain image could be extended, by duplicating the rightmost and leftmost strips of the input image and concatenating them to its left and right side, respectively. From the concatenated image, the network would predict A-line coordinates for a wider image, from which the original sized image and (predicted) A-line coordinates vector can be retrieved.

Another limitation of ACE-Net is related to the suitability of the A-line encoding scheme for all vascular structures, such as those obtained from intracoronary or intracranial IVUS/OCT scans. While it performed well for the structures we investigated, more complex topologies, such as those with holes, might need more than two boundary vectors. However, most vascular structures have layer-like configurations that fit our representation. To extend ACE-Net’s applicability to more anatomical structures in US segmentation tasks, alternative or modified encoding schemes could be explored.

ACE-Net’s best performance requires a specific training process: the bottom and top models are trained separately and then frozen and unfrozen. Moreover, annotations have to be re-encoded into pixel-wise and A-line coordinates. The training process, which involves weight switching and module unfreezing, must be manually controlled or automatically scheduled. Both options have drawbacks: the former needs more human attention, while the latter adds more hyperparameters for the model.

### Future work

As ACE-Net outputs boundary coordinate vectors, which are convenient for annotation computer interfaces, future research could investigate the introduction of iterative human correction in the pipeline of ACE-Net. By doing so, only minimal correction from experts would be required, significantly reducing the annotation burden and opening up the possibility for a human-in-the-loop mechanism [12].

Future work could also encompass using different feature extraction mechanisms instead of the current backbone module to remove the need of an additional pixel-wise loss for training. Extending the feature visualization highlighting areas of activation for different contours could support the explainability of ACE-Net and improve the understanding of its variants.

Additionally, the integration of a transformer for A-line feature extraction and A-line coordinates encoding in ACE-Net will be explored. For instance, the transformer could be utilized to process individual A-lines as sequential input by applying positional encoding to capture their spatial relationships. This integration has the potential to enhance the representation learning capabilities of ACE-Net and further improve its segmentation performance.

Finally, because ACE-Net was inspired by side-viewing images, ACE-Net and the proposed A-line-based encoding scheme could be directly adapted to other side-viewing imaging modalities, such as endoscopic OCT imaging.

### Supplementary information

Supplementary material includes more examples of the output of ACE-Net for the IVUS-plaque/calcium dataset and of the mixed with synthetic training set (IVUS-lumen dataset training images and generated synthetic images). A table of the ACE-Net hyperparameters used in all experiments is also included.

## Supplementary Information

Below is the link to the electronic supplementary material.

Supplementary file 1 (zip 69403 KB)
